# Next-Generation Sequencing Reveals Continued Circulation of Rare HIV-1 Subtypes in the Democratic Republic of the Congo and Refines the Estimate of the Emergence Dates of Three Subtypes

**DOI:** 10.3390/v18020268

**Published:** 2026-02-21

**Authors:** Mark Anderson, Gregory S. Orf, Vera Holzmayer, Ana Olivo, Barbara J. Harris, Michael G. Berg, Guixia Yu, Asmeeta Achari, Scot Federman, Charles Y. Chiu, Linda James, Samuel Mampunza, Gavin A. Cloherty, Mary A. Rodgers

**Affiliations:** 1 Discovery Research, Abbott Laboratories, Abbott Park, IL 60064, USA; mark.anderson6@abbott.com (M.A.); gregory.orf@abbott.com (G.S.O.); gavin.cloherty@abbott.com (G.A.C.); 2 Department of Laboratory Medicine, University of California San Francisco, San Francisco, CA 94158, USA; 3 Faculté de Médecine, Université Protestante au Congo, Kinshasa B.P. 4745, Democratic Republic of the Congo

**Keywords:** human immunodeficiency virus 1, next-generation sequencing, HIV-1 genotypes, HIV-1 subtypes, Bayesian inference of phylogeny

## Abstract

HIV-1 diversified for decades within the Democratic Republic of the Congo (DRC) before spreading globally in the early 1980s. Thus, the DRC is home to some of the most ancestral and diverse HIV-1 strains. Recent serosurveys conducted from 2017 to 2019 in Kinshasa, DRC, indicated high prevalence of HIV-1, yet sequence data is lacking from this period. Given the history of circulating rare HIV-1 subtypes in the DRC, a viral whole-genome sequencing study was conducted to determine current diversity in the greater Kinshasa area. Next-generation sequencing (NGS) through metagenomic and target enrichment methods was conducted on 197 specimens collected from 2017 to 2019. A large array of HIV subtypes (A, B, C, D, F1, G, H, J, and K), circulating recombinant forms (CRF01_AE, CRF02_AG, CRF05_DF, CRF11_cpx, CRF13_cpx, CRF25_cpx, CRF 45_cpx, and CRF92_C2U), unique recombinant forms, and unclassifiable sequences were observed, with many branching in basal positions within, or outside of, many subtypes on phylogenetic trees. Incorporating these new sequences into Bayesian inference of phylogeny pushes back the dates of the most recent common ancestors of HIV-1 group M and the rare subtypes G, H, and J by between 3 and 7 years each. The DRC continues to harbor diverse and rare HIV-1 subtypes that could challenge diagnostic tests, treatments, and vaccines. In addition to shifting subtype emergence dates, the sequences from our study are evidence that rare strains continue to circulate and should be regularly monitored.

## 1. Introduction

Human immunodeficiency virus 1 (HIV-1) originated in the Congo River Basin in the early 1900s through interspecies transmission of simian immunodeficiency virus (SIV) to humans before spreading and diversifying in the region [1]. Consequently, the Congo Basin, including the Democratic Republic of the Congo (DRC), contains the greatest HIV-1 group M diversity in the world [2]. The earliest sampled HIV-1 sequences date back to 1959 and 1960, both originating from Kinshasa in the DRC, before the onset of the HIV-1 global epidemic [3,4]. These two sequences had already diverged significantly from their common ancestor, sharing only 88% identity with each other. Contemporary analyses show the 1959 specimen branching with subtype D references and the 1960 isolate branching with the contemporary subtype A [3,4,5]. However, further analysis of these early genomes indicated that HIV-1 had been circulating much earlier, as prior estimates place the origins of HIV-1 group M in the early 1900s (1884–1924) [1,4,6]. A 2017 study of HIV-1 diversity in the DRC demonstrated that specimens collected between 2001 and 2003 continued to harbor a vast array of diverse HIV-1 pure subtypes as well as recombinants [7]. This was followed by the discovery of the third known sequence of a full-length HIV-1 group M, subtype L, in the DRC from a specimen collected in 2001 [8]. Our recent prevalence studies, conducted between 2017 and 2019 in the greater Kinshasa area, showed a high rate of HIV-1 infection, as well as a large proportion of potential elite controllers capable of suppressing viral load without medication [9,10]. Given the minimal HIV-1 genomic sequencing data available from the DRC after 2003, here, we sought to generate sequences from viremic cases in the 2017–2019 prevalence study. Whole-genome HIV-1 sequencing affords an opportunity to better understand the genetic landscape of the epidemic in an epidemiologically important region and evaluate the performance of diagnostic tests with currently circulating strains.

As of late 2025, the Los Alamos National Laboratory (LANL) sequence database recognizes four HIV-1 groups (M, N, O, and P), ten HIV-1 group M subtypes, >170 HIV-1 group M circulating recombinant forms (CRFs), and numerous unique recombinant forms (URFs), which are all defined based on phylogenetic relatedness [11]. This massive diversity has posed a detection challenge for diagnostic assays, particularly among molecular tests that were initially developed based on a single target [12,13,14,15]. Surveillance and continual testing of diverse HIV-1 specimens are crucial to ensuring that diagnostic tests can detect this vast sequence expanse. Due to how widespread and diverse HIV-1 group M has become, considerable work has been undertaken to understand the emergence, evolution, and epidemiological characteristics of the different subtypes and CRFs using multiple phylogenetic methods [16,17,18,19,20]. Unfortunately, these approaches can be severely limited by the available number of sequences. In cases of widespread subtypes like subtype B, there is an overabundance of sequencing information that requires complex downsampling steps [21]. However, the opposite is true for less prevalent subtypes such as subtype H, as there have been too few sequences collected to perform robust molecular dating analyses [22]. Thus, Kinshasa and its surrounding districts represent an ideal location to address these knowledge gaps.

Here, we report complete and near-complete HIV-1 genomes and an accompanying phylogenetic analysis for 197 specimens collected between 2017 and 2019 from the greater Kinshasa area. We confirm diagnostic detection with 100% sensitivity using a suite of serological, rapid, and molecular assays for these diverse strains. Importantly, we have doubled the number of HIV-1 subtype H sequences from the DRC, which likely emerged locally in the DRC early in the epidemic. Including the new H sequences and other rare strains found in this study in a time-dependent Bayesian inference of phylogeny refined the estimate of the appearance of the last common ancestor of HIV-1 group M to earlier than previous estimates.

## 2. Materials and Methods

### 2.1. Specimen Collection

Ethics approval and consent to participate were obtained from the Université Protestante au Congo (UPC) Institutional Review Board (CEUPC-027) [9,10]. Deidentified plasma specimens (*n* = 197) were collected through an HIV prevalence study between March 2017 and February 2019 from 31 health facilities in and around Kinshasa (Tables S1 and S2) [9]. Patients were provided an HIV rapid test (RDT) free of charge, and the leftover plasma sample was stored for further characterization. Seropositivity was confirmed following the DRC national testing algorithm before subsequent viral testing and sequencing.

### 2.2. Testing

Specimens were screened for evidence of HIV infection using the ARCHITECT HIV Ag/Ab Combo assay (Abbott GmbH, Weisbaden, Germany). Specimens reactive for HIV Ag/Ab were then screened for viral load using the RealTi*m*e HIV-1 assay (Abbott Molecular, Des Plaines, IL, USA). When volume allowed, a subset of specimens (*n* = 161) was further tested for HIV viral load using the Alinity m HIV-1 assay (Abbott Molecular, Des Plaines, IL, USA). For rapid HIV detection, the subset was tested with the Determine HIV-1/2 Ag/Ab Combo RDT assay (Abbott Diagnostics Scarborough Inc., Scarborough, ME, USA). The subset specimens were also tested with the Investigational Use Only (IUO) Alinity i HIV Ag/Ab Combo Next assay (Abbott Ireland Diagnostics Ltd, Sligo, Ireland). All tests were performed according to the manufacturer’s instructions.

### 2.3. Next-Generation Sequencing

Specimens were processed for next-generation sequencing (NGS) on the Illumina short-read platform using either a primer-spiked metagenomic (MSSPE, metagenomic sequence with spike primer enrichment) or target enrichment procedure (xGen) as previously described [23,24]. For the metagenomic approach, a panel of HIV-specific reverse primers was spiked into the random primer metagenomic pool at a 10:1 ratio and sequenced on an Illumina NextSeq (Illumina, San Diego, CA, USA). For the target enrichment method, a non-redundant biotin-labeled HIV-xGen probe set was synthesized to tile across the genomes of all HIV-1 (groups M, N, O, and P) and HIV-2 (A and B) viruses. Nextera barcoded metagenomic libraries were hybridized to the xGen probes in pools, and unbound sequences were washed away. Captured viral cDNA was amplified by Illumina adaptor primers and sequenced on an Illumina MiSeq instrument (Illumina, San Diego, CA, USA).

### 2.4. Sequence Analysis

Illumina NGS FASTQ files were processed using a custom, in-house, automated pipeline to generate consensus genomes based on a well-curated database of HIV reference sequences sourced from GenBank. Specimens with greater than 90% HIV genome coverage were manually reviewed and corrected for frameshifts and base calling using Integrated Genomics Viewer v2.16.2 (https://igv.org/) and modified, if necessary, in BioEdit v7.2.5 (https://bioedit.software.informer.com/7.2/, accessed on 15 July 2025). Final genomes were submitted to GenBank (Supplementary Information, Table S1).

### 2.5. Maximum Likelihood Phylogenetic and Recombination Analysis

Sequences were aligned (using mafft) with 467 curated references (Supplementary Information, Table S3) encompassing all HIV-1 group M subtypes and 133 CRFs to generate maximum likelihood trees using IQ-TREE v2.2.2.6 and ModelFinder to choose the best-fit model [25,26]. Bootstrap values were calculated with IQ-TREE’s integrated ultrafast bootstrap approximation with 1000 replicates [27]. Resulting phylogenetic trees were visualized with FigTree v1.4.3 software (https://tree.bio.ed.ac.uk/software/figtree/, accessed on 15 July 2025) and manually annotated. Subtyping was completed through a manual phylogenetic inference process whereby if a study sequence branched with references with bootstrap support of at least 70, then it was classified as belonging to the same subtype as the reference. Sequences branching basal to reference subtypes/CRFs were analyzed for evidence of recombination using Simplot v3.5.1 (https://sray.med.som.jhmi.edu/SCRoftware/SimPlot/, accessed on 15 July 2025) for similarity plot and bootscanning analyses as previously described [7,28]. The outgroup in these analyses was a simian immunodeficiency virus (SIV_CPZ) with Genbank accession number X52154.

### 2.6. Estimation of Temporal Signal

Curated multiple sequence alignments (MSAs) of HIV-1 group M (including CRFs but excluding URFs) were downloaded from the LANL HIV sequence database on 6 May 2025. These included a whole-genome alignment (3310 sequences), as well as individual *gag*, *pol*, and *env* gene alignments (3310, 4293, 2809, and 4690 sequences, respectively). This dataset was called Dataset ML-1. A custom Augur pipeline (NextStrain platform) [29] was written to expedite time-calibrated maximum likelihood analyses on these datasets, while also incorporating the 197 new sequences generated in this study for comparison (the dataset incorporating the LANL sequences plus the new sequences was called Dataset ML-2). Outlier sequences that violated the inferred molecular clock (i.e., those that deviated more than 4 interquartile ranges from the root-to-tip versus sampling time regression, as calculated by the TreeTime module of Augur’s Refine tool. The resulting NextStrain JSON output file was inspected using Auspice [29]. A custom *R* script was written to extract the root-to-tip distances for each sequence from the JSON output file and produce publication-quality images of the molecular clock signal.

### 2.7. Reconstruction of Bayesian Phylogenies

All sequences corresponding to the *env* gene for the rare subtypes G, H, J, and L were downloaded from the LANL database on 10 June 2025; this dataset contained 166 sequences. The “NextStrain” *env* gene alignment was also downloaded from the LANL database on the same date; this dataset contained 3775 sequences collected between 1983 and 2021 and has already been pre-downsampled from the full database using time and geography filters (https://nextstrain.org/groups/LANL-HIV-DB, accessed on 10 June 2025). Due to the high representation of common subtypes A, B, and C, any sequences collected after 1990 for these subtypes were further randomly downsampled by 50%, 95%, and 95%, respectively. The subtype G, H, J, and L sequences were added back to the alignment and deduplicated, resulting in a representative dataset enriched in subtypes G, H, J, and L, composed of 568 *env* sequences. This dataset is called Dataset BI-1, and accessions can be found in the Supplementary Information, Table S4. The 28 sequences collected in this study, putatively assigned to any of these four subtypes, were then added to produce Dataset BI-2. Maximum likelihood trees for each dataset were calculated using IQ-TREE v2.1.3 [25] with automatic model selection using the ModelFinder [26] module with bootstrap support calculated using 1000 replicates of UFBoot2 [27]. Presence of temporal signal was confirmed with both TempEst v.1.5.3 [30] and TreeTime v.0.8.5 [31] using re-rooting to either (1) minimize residuals, or (2) maximize correlation, in a root-to-tip versus sampling time regression.

To reconstruct a time-calibrated evolutionary history of the above datasets, we utilized Bayesian inference of phylogeny using the Markov chain Monte Carlo (MCMC) framework as implemented in BEAST v.1.10.5 [32], accelerated by the BEAGLE v.4.0.0 library [33]. For each dataset, we used a GTR+4Γ substitution model, an uncorrelated relaxed clock model [34] with lognormal distribution, and a Bayesian Skyride non-parametric coalescent tree prior. MCMC analysis was performed in triplicate for each dataset and tree prior for 2.5 × 10^8^ chains, sampling every 2.5 × 10^4^ chains for both log and tree files. At least ten percent burn-in was removed from all logs and tree files, and replicate runs were combined using LogCombiner v.1.10.4 [32]; calculations were considered complete when the combined logs showed effective sample size (ESS) values for all parameters greater than 250, as assessed by Tracer v.1.7.2 [35]. Maximum clade credibility (MCC) trees were generated using TreeAnnotator v.1.10.4 [32] from the tree files resulting from the combination of replicates. MCC trees were visualized using the *ggtree* [36] and *ggplot2* [37] packages for *R*.

## 3. Results

### 3.1. Subtype Classification

HIV-1-positive specimens were collected between March 2017 and February 2019 as part of a previously reported HIV prevalence survey in the DRC [9]. Specimens with sufficient viral load and remaining volume (*n* = 197) from Kinshasa and the surrounding area of the DRC were selected for further testing and genome sequencing using a combination of primer-spiked metagenomic and/or target enrichment NGS approaches. Summarized demographic data, including age, sex, and collection site, were reported when available (as can be seen in Table S2).

**Figure 1:**
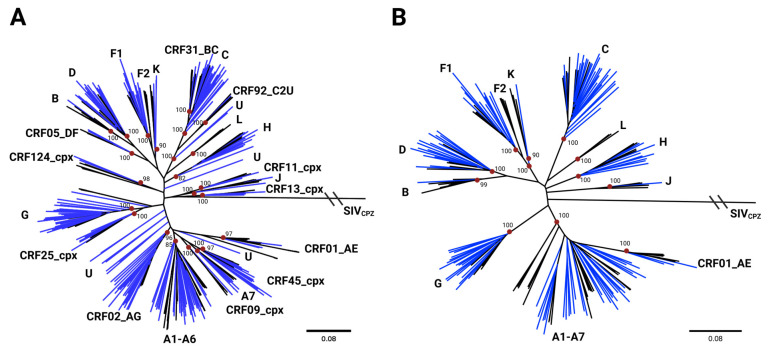
IQ-TREE-generated maximum likelihood trees containing references and (**A**) all study sequences or (**B**) study sequences that were confirmed to be pure subtypes (including CRF01_AE) based on recombination analysis using SimPlot. Black lines indicate reference sequences used for subtype classification. Blue lines indicate sequences generated in this study. Surrounding labels indicate subtype/CRF locations on the tree. Red dots and neighboring values indicate important branch points on the tree and corresponding bootstrap values. The outgroup in both panels is SIV_CPZ_. Angled marks indicate that the branch length was truncated for visualization. Scale bars are included to show branch length diversity.

**Figure 2:**
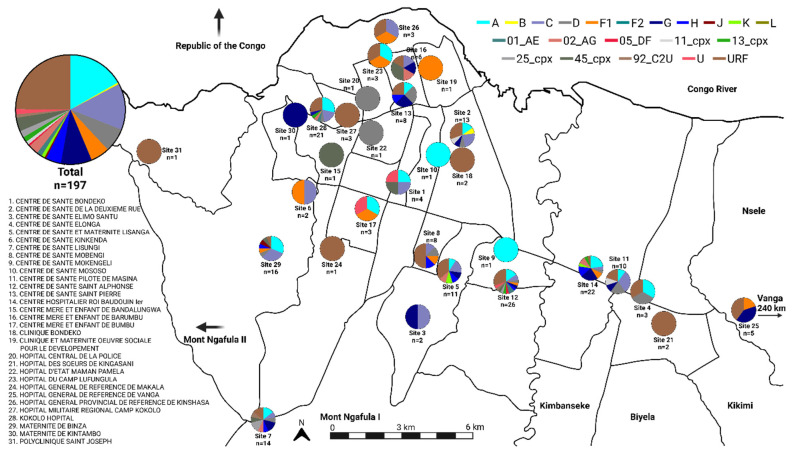
Map of 31 collection sites in Kinshasa and surrounding districts in the DRC. Pie charts indicate HIV subtype classification percentages obtained from NGS results for specimens collected from each site. Legends indicate collection site locations and the color associated with HIV subtypes.

Initial subtype classification was based on maximum likelihood phylogenetic trees in which full-genome sequences were compared to a curated HIV-1 reference dataset, containing 467 representatives of the pure HIV-1 subtypes and 133 CRFs (Supplemental Information, Figure S1). After removing unrelated sequences from the reference dataset, classifications were assigned by analysis of ML trees consisting of all study sequences or those classified as pure subtypes (Figure 1A and Figure 1B, respectively). The ML tree restricted to pure subtypes revealed substantial diversity among the newly sequenced HIV isolates, many of which branched near the roots of their respective subtype groups (Figure 1B). In total, the new sequences were classified as pure subtypes (A, B, C, D, F1, G, H, J, and K), CRFs (CRF01_AE, CRF02_AG, CRF05_DF, CRF11_cpx, CRF13_cpx, CRF25_cpx, CRF45_cpx, and CRF92_C2U), unclassifiable (U) groups, and URFs (Table 1). A comparison between the subtype classifications and the geographic location of collection sites did not suggest geographic localization; indeed, most subtypes and CRFs appeared to be well-dispersed across Kinshasa and the surrounding regions (Figure 2). Notably, a single subtype B was found and was determined to likely be a re-introduction from outside the DRC (Supplemental Information, Figure S2).

### 3.2. Diagnostic Performance Against Rare Strains

Given the rarity and extensive subtype diversity observed in this study, diagnostic assay performance was assessed with a suite of serological Ag/Ab, molecular, and rapid lateral flow Ag/Ab assays on samples with sufficient volume (Supplemental Information, Table S1). Each diagnostic test detected all tested strains with 100% sensitivity (Table 1). Serologic testing (*n* = 197) was performed using the HIV Ag/Ab Combo assay on the Abbott ARCHITECT automated platform with signal to cutoff (S/CO) values ranging from 17.65 to 1164 (median, 701.6 S/CO). Additional serologic testing (*n* = 161) was conducted on 1:10 diluted samples using the Investigational Use HIV Ag/Ab Combo Next assay on the automated Abbott Alinity i platform with reported S/CO values ranging from 30.04 to 496 (median, 222.3 S/CO). Molecular viral load (VL) testing was conducted using the Abbott Molecular RealTi*m*e HIV-1 Viral Load (*n* = 197) and Abbott Molecular Alinity m HIV-1 Viral Load (*n* = 161) assays. Despite testing being conducted on different days and aliquots with separate freeze–thaw cycles, comparison of VL testing demonstrated good concordance (R^2^ = 0.844), and no specimens yielded results differing by more than 1 log copies/mL (range −0.67 to 0.83 and median 0.08, log copies/mL) between the assays (Supplemental Information, Figure S3). Lastly, rapid diagnostic testing (*n* = 161) was conducted using the Abbott Determine HIV-1/2 Ag/Ab Combo RDT.

### 3.3. Time-Dependent Phylogenetic Analyses

**Figure 3:**
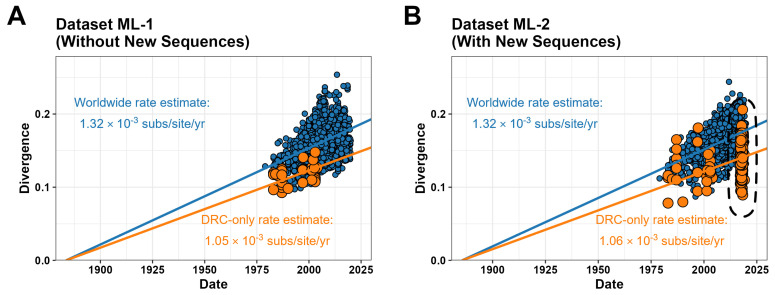
Maximum likelihood-based strict molecular clock analysis (root-to-tip distance versus sampling date using the NextStrain pipeline) of complete HIV-1 genomes from the LANL reference dataset alone (**A**) and the LANL reference dataset combined with the 197 new sequences recovered in the current study (**B**). Linear regressions with accompanying clock rate estimates are shown for sequences from all countries (including the DRC; blue) and the DRC alone (orange). Datapoints for new DRC sequences from this study are circled with a dashed line.

To investigate the overall quality of sequences generated in this study, we compared our 197 new full-genome sequences to 3310 curated group M sequences (without URFs) from the LANL database using ML-based root-to-tip regression with a simplified strict clock model implemented with the NextStrain pipeline (Figure 3). The curated dataset alone (Dataset ML-1; Figure 3A) indicated an evolutionary rate estimate of 1.32 × 10^−3^ subs/site/yr with a time of most recent common ancestor (tMRCA) in the year 1883, consistent with prior estimates using those methods [1,4,6]. When sequences from the DRC were isolated, a slower evolutionary rate of 1.05 × 10^−3^ subs/site/yr was estimated. When the new sequences were included (Dataset ML-2; Figure 3B), the worldwide and DRC-only evolutionary rates largely remained the same (1.32 × 10^−3^ and 1.06 × 10^−3^ subs/site/yr, respectively, with a tMRCA in the year 1885). Importantly, none of the 197 new individual sequences in this analysis deviated from the clock signal regression by more than three interquartile ranges (i.e., none were outliers). This provides an additional metric showing that systemic issues related to sequencing quality in our 197 new genomes were unlikely to be present. The above behavior is maintained even in individual genes (for example, *env*; Supplemental Information, Figure S4).

**Figure 4:**
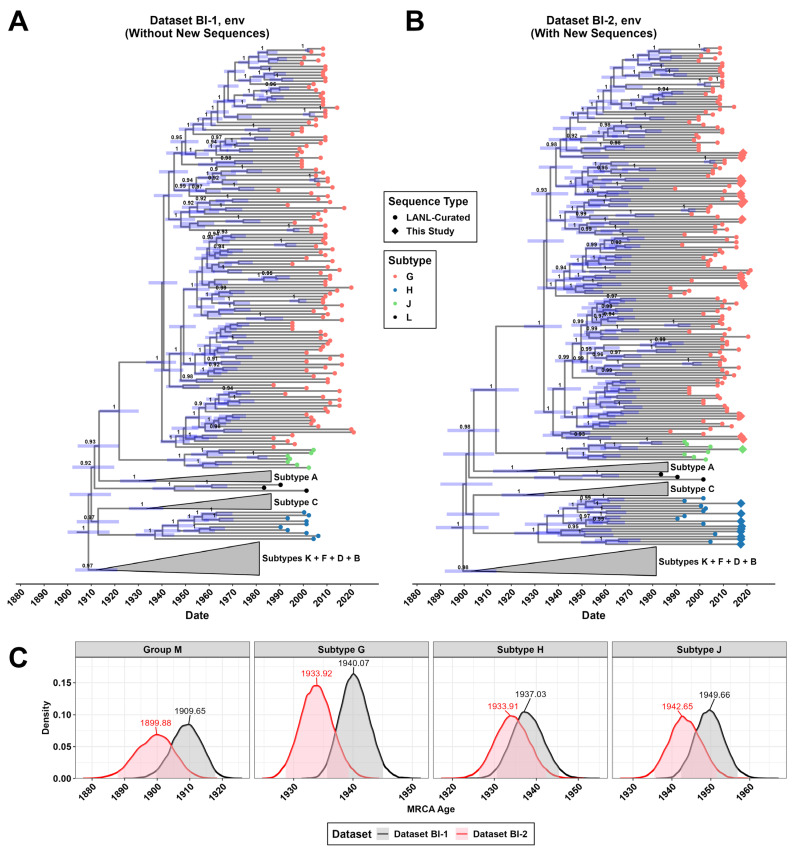
Bayesian inference of phylogeny and demographic history for HIV-1 group M with focus on subtype G, H, and J. (**A**) Phylogeny of the *env* gene from the LANL reference dataset alone (Dataset BI-1). (**B**) Phylogeny of the *env* gene from the LANL reference dataset plus the 28 new sequences generated in this study (Dataset BI-2). For each maximum clade credibility tree, an uncorrelated local clock with an underlying lognormal distribution was assumed with a coalescent Bayesian Skygrid tree prior. Tips are colored by subtype with shapes denoting the source of the sequence. Posterior probabilities and 95%-highest probability densities (HPDs) for height are shown at each node as a number (only when >0.9) and a light blue range bar, respectively. (**C**) Node age density distributions for the MRCA of subtypes G/H/J and all of group M, stratified by dataset. Median ages are labeled, and the 95%-HPD is shaded.

**Figure 5:**
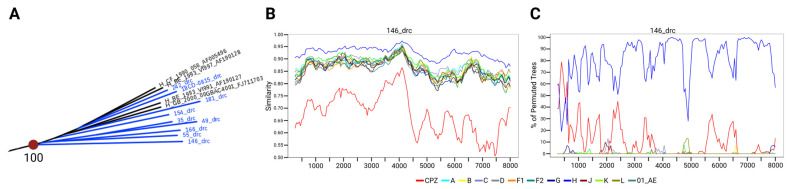
(**A**) Subtype H branch of the ML tree from Figure 1B. The red dot and number indicate the basal node of the branch with bootstrap value. (**B**) Similarity plot of the genome from specimen 146 (most basal H on the tree) comparing similarity between subtypes. (**C**) Bootscan of the genome from specimen 146 showing no evidence of recombination between subtypes. For SimPlot analysis in (**B**,**C**), all pure H subtypes from this study were included as references, and a window of 500 bp and step of 50 bp were used. For bootscanning, 1000 bootstrap replicates were used. To more clearly indicate sequences generated in this study, “_drc” was added to the specimen IDs in the figure. Subtype reference consensus sequences were used for SimPlot and Bootscan analyses.

Notably, the H clade, which is restricted to the Congo Basin and has doubled in size with the addition of these new sequences, is closer to the MRCA for group M than other subtypes (Figure 1A,B and Figure 4A). To determine whether the recently sequenced H genomes could provide insight into the early evolution of HIV-1, this branch was examined in more depth. A closer analysis of the H branch depicted in Figure 1 revealed that seven of the nine pure subtype H genomes branched basal to the LANL reference sequences with high bootstrap values (Figure 5A). Specimen 146, which branched the most basal to subtype H references on this ML tree, was further analyzed by SimPlot recombination analysis software using DRC and reference subtype H sequences (Figure 5B,C). The analysis did not reveal evidence of recombination, supporting this genome’s classification as pure subtype H. Recombination analysis of other new genomes belonging to the same H branch on this tree also confirmed pure subtype H classifications (Supplemental Information, Figure S5).

Next, we evaluated whether the inclusion of the new sequences would alter tMRCA estimates of the origin of subtype H. To produce a more robust dataset amenable to Bayesian inference of phylogeny, we aligned all group M non-recombinant *env* sequences available through the curated LANL database then downsampled the non-subtype G, H, J, and L entries, resulting in 568 sequences (Dataset BI-1). We also produced a second dataset that includes the 28 sequences belonging to these subtypes that were generated in this study (G: 18; H: 9; J: 1), totaling 596 sequences (Dataset BI-2).

Bayesian inference of phylogeny for these two datasets was performed using the more flexible uncorrelated local clock with underlying lognormal distribution (UCLN). The maximum clade credibility (MCC) trees for both datasets using the UCLN clock model and non-parametric Bayesian Skyride coalescent prior are presented here (Figure 4). Each tree showed strong statistical support for the early branching and separation of the subtypes G, H, and J, though the timing of each branching event differed when adding the newly acquired sequences. The new sequences pushed back the median estimate for the *env* gene tMRCA for subtype H from 1937 (95%-HPD: 1930.56–1945.45) to 1933 (95%-HPD: 1926.37–1942.33), the tMRCA of subtype G from 1940 (95%-HPD: 1935.63–1945.07) to 1933 (95%-HPD: 1928.65–1939.28), and the tMRCA of subtype J from 1949 (95%-HPD: 1942.38–1956.89) to 1942 (95%-HPD: 1935.48–1951.49). The analysis also pushed back the emergence of the group M from 1909 (95%-HPD: 1899.72–1917.84) to 1899 (95%-HPD: 1887.89–1910.31).

## 4. Discussion

Since its discovery in 1983, HIV has continued to test the limits of the global health community due to its genetic diversity, rapid mutation rate, and ability to recombine with other HIV strains. The first FDA-approved test for screening blood for the presence of HIV antibodies (HTLV-III, Abbott Laboratories) was released 40 years ago in 1985 and immediately improved the safety of the blood supply before the full diversity and scope of HIV sequences were comprehended [38,39,40]. Due to the high HIV-1 genetic variability that has been revealed since then, diagnostic assays for the detection and monitoring of HIV have been continually challenged by the ongoing evolution of this virus. Nowhere has this diversity been more apparent than in the Congo River Basin, where the HIV epidemic began [41,42]. Within this region, the prevalence of HIV has remained high, with both highly diverse and ancestral HIV-1 strains commonly observed [9,43].

HIV genomes are difficult to assemble and characterize due to high inter- and intra-host genetic diversity, and widely variable viral loads within infected individuals [44,45,46]. These challenges have been significantly offset in recent years through the development of targeted viral NGS methods combined with advanced computational analyses [23,24,44,47]. Finalizing consensus genomes of high quality is of critical importance for the success of HIV drug resistance testing [46,48], molecular epidemiology studies [21,49,50], and infection timing when tracking transmission networks [51,52]. One measure of sequence quality is to compare the evolutionary rate of newly acquired sequences against a large, curated database; sequences with an unexpectedly large number of mutations (i.e., stemming from poor sequence quality rather than from true evolutionary forces) will appear to violate the molecular clock signal.

An important result of our study is the wealth of new representatives from rarer HIV-1 group M subtypes. It is currently believed that subtypes A, B, C, and CRFs 01_AE and 02_AG comprise 82.0% of worldwide group M infections and 36.5% of central African group M infections [53]. Within the 197 genomes presented here, 35.6% (70/197) were classified as belonging to the above-mentioned common subtypes and CRFs (Table 1). A total of 78 of our 197 sequences (39.6%) were classified as recombinants (i.e., CRFs and URFs combined), slightly lower than previous prevalence estimates of 46.8% in central Africa [53]. The rare subtypes G, H, J, and K comprised 15.2% (30/197) of our sequences, which is 50% higher than previous estimates of their combined prevalence in central Africa, which stood at 10.2% [53]. Of particular interest were the nine new full- or nearly full-genome representatives from subtype H, which now nearly doubles the known number of genomes available (only ten previously).

Beyond subtype H, our findings also indicate the recent and ongoing presence of rare HIV subtypes G, J, and K, as well as subtypes A–F1, CRFs, unclassifiable subtypes, and URFs, that challenge diagnostic tests (Figure 1 and Figure 2). In each case, we found 100% sensitivity for all diagnostic tests used (Table 1), highlighting the strong performance of current and next-generation tests in the detection of highly diverse HIV-1 subtypes. Furthermore, ML phylogenies suggest that many of the new sequences branch at basal positions within respective subtypes (Figure 1). Considering that the DRC is the original epicenter of HIV emergence, this indicates that the HIV strains currently circulating in the DRC have accumulated fewer mutations relative to the MRCAs of those subtypes than strains that have spread to neighboring countries and outside of Africa. This is consistent with the geographical restriction of subtypes J, K, and H being the product of long-term local diversification, rather than the product of international export and re-import. The major exception seems to be subtype B, which has rarely been seen in the DRC despite its predominance in the US and Europe [43]. Notably, we detected only a single subtype B sequence in this study, which was most related to other contemporary references from outside Africa with strong bootstrap support. This suggests that this sequence is the product of a re-introduction to the DRC well after subtype B had undergone diversification outside of Africa.

We were particularly interested in the nine near-complete subtype H sequences, which greatly expand the diversity of this rare subtype that is resident in the Congo Basin and bears relevance to the early history of the diversification of HIV-1. Only a few near-full-length genomes of subtype H have been previously reported, and only ten are listed in the curated LANL database [7,11,54,55]. Due to this deficiency, tMRCA estimates for subtype H are lacking [22]. The inclusion of these nine new subtype H sequences to the dataset altered tMRCA prediction via BI for the *env* gene from this subtype, pushing back the median estimate by just over 3 years (from early 1937 to late 1933). Including the new 28 sequences across subtypes G, H, and J also pushes back the median estimate for the tMRCA of the *env* gene of all of group M by 10 years (from mid-1909 to late 1899). Additional studies using our new basal G, H, and J sequences may be useful in understanding whether they may have reduced fitness compared to subtypes that spread globally [56].

In conclusion, herein we report on the high genomic sequence diversity and 100% diagnostic sensitivity of recently circulating HIV-1 strains from the greater Kinshasa area in the DRC. Our study nearly doubles the number of subtype H genomes available to enable refined evolutionary history studies. Since socio-economic factors were the primary drivers of the global expansion of the more common group M subtypes A1, C, and D [56], it remains possible that any of the rarer subtypes that are geographically restricted today may have equal potential to spread globally, especially in a highly connected global community with increasing rates of travel. Thus, future studies should incorporate the sequences recovered in this study to better refine tMRCA calculations for other HIV-1 subtypes and to define potentially new CRFs. Given the observed impact of adding nine new subtype H sequences to our analyses, this study highlights the continued importance and relevance of HIV surveillance in the DRC and should spur further HIV monitoring in this region.

## Figures and Tables

**Table 1:** Summary of sequenced subtype classifications and diagnostic test results.

Subtype or CRF/URF	Number of Sequences	ARCHITECT HIV Ag/Ab Combo Tested/Detected (%)	RealTi*m*e HIV-1 Viral Load Tested/Detected (%)	Alinity i HIV Ag/Ab Combo Next Tested/Detected (%) ^a^	Alinity m HIV-1 Viral Load Tested/Detected (%) ^a^	Determine HIV-1/2 Ag/Ab COMBO RDT Tested/Detected (%) ^b^
A	33	33/33 (100)	33/33 (100)	29/29 (100)	29/29 (100)	29/29 (100)
B	1	1/1 (100)	1/1 (100)	1/1 (100)	1/1 (100)	1/1 (100)
C	27	27/27 (100)	27/27 (100)	19/19 (100)	19/19 (100)	19/19 (100)
D	14	14/14 (100)	14/14 (100)	14/14 (100)	14/14 (100)	14/14 (100)
F1	11	11/11 (100)	11/11 (100)	10/10 (100)	10/10 (100)	10/10 (100)
G	18	18/18 (100)	18/18 (100)	15/15 (100)	15/15 (100)	15/15 (100)
H	9	9/9 (100)	9/9 (100)	3/3 (100)	3/3 (100)	3/3 (100)
J	1	1/1 (100)	1/1 (100)	1/1 (100)	1/1 (100)	1/1 (100)
K	1	1/1 (100)	1/1 (100)	N/A	N/A	N/A
CRF01_AE	3	3/3 (100)	3/3 (100)	3/3 (100)	3/3 (100)	3/3 (100)
CRF02_AG	6	6/6 (100)	6/6 (100)	6/6 (100)	6/6 (100)	6/6 (100)
CRF05_DF	1	1/1 (100)	1/1 (100)	1/1 (100)	1/1 (100)	1/1 (100)
CRF11_cpx	2	2/2 (100)	2/2 (100)	2/2 (100)	2/2 (100)	2/2 (100)
CRF13_cpx	3	3/3 (100)	3/3 (100)	3/3 (100)	3/3 (100)	3/3 (100)
CRF25_cpx	4	4/4 (100)	4/4 (100)	4/4 (100)	4/4 (100)	4/4 (100)
CRF45_cpx	8	8/8 (100)	8/8 (100)	8/8 (100)	8/8 (100)	8/8 (100)
CRF92_C2U	2	2/2 (100)	2/2 (100)	1/1 (100)	1/1 (100)	1/1 (100)
U	3	3/3 (100)	3/3 (100)	2/2 (100	2/2 (100	2/2 (100
URF	50	50/50 (100)	50/50 (100)	39/39 (100)	39/39 (100)	39/39 (100)
Total	197	197/197 (100)	197/197 (100)	161/161 (100)	161/161 (100)	161/161 (100)

^a^ Specimens were tested at a 1:10 dilution in normal human plasma. ^b^ All samples were positive for HIV antibody only.

## Data Availability

All finalized sequences from this study have been deposited in GenBank and accession numbers are reported in Supplementary Information, Table S1.

## Supplementary Materials

The following supporting information can be downloaded at https://www.mdpi.com/article/10.3390/v18020268/s1, Table S1: Serologic, molecular, and rapid diagnostic testing results by sample; Table S2: Summary of participant demographics and study collection sites; Table S3: HIV-1 group M references used for phylogenetic trees presented in Figure 1 of the main text; Table S4: HIV-1 group M references used for phylogenetic trees presented in Figure 5 of the main text; Figure S1: Maximum likelihood tree of DRC specimens along with 467 GenBank references; Figure S2: Maximum likelihood phylogeny performed via the Augur pipeline (NextStrain), zoomed to the branch of subtype B of which the HIV-1 full genome from 18CD-0068 belongs; Figure S3: Comparison of results obtained from RealTi*m*e HIV-1 (x-axis) and Alinity m viral load (y-axis) testing; Figure S4: Molecular clock analysis (root-to-tip distance versus sampling date) of all HIV-1 env gene sequences from the LANL reference dataset alone and the LANL reference dataset combined with our 197 new sequences; Figure S5: SimPlot and Bootscan of 8 additional subtype H specimens identified in the study.
