# Supplementary material for: Next-Generation Sequencing Reveals Continued Circulation of Rare HIV-1 Subtypes in the Democratic Republic of the Congo and Refines the Estimate of the Emergence Dates of Three Subtypes
Source: Viruses. 2026 Feb 21;18(2):268. doi: 10.3390/v18020268 (PMC12945268; doi:10.3390/v18020268)

Supplementary Information for:

Next Generation Sequencing Reveals Continued Circulation of Rare HIV-1 Subtypes in The Democratic Republic of the Congo and Refines the Estimate of the Emergence Dates of Three Sub-types

Mark Anderson[1], Gregory S. Orf[1], Vera Holzmayer[1], Barbara Harris[1], Michael G. Berg[1], Guixia Yu[2], Asmeeta Achari[2], Scot Federman[2], Charles Y. Chiu[2], Linda James[3], Samuel Mampunza[3], Gavin A. Cloherty[1], Mary A. Rodgers[1,*]

[1] Infectious Disease & Discovery Research, Abbott Laboratories, Abbott Park, IL, USA
[2] Department of Laboratory Medicine, University of California San Francisco, San Francisco, CA, USA
[3] Université Protestante au Congo, Kinshasa, Democratic Republic of the Congo
* Correspondence: mary.rodgers@abbott.com

Supplemental Table S1: Serologic, molecular, and rapid diagnostic testing results by sample. Specimen collection date and subtype classification for each sample are included.

Specimen Identifier	Genbank Accession Number	Collection date	ARCHITECT HIV Ag/Ab Combo (S/CO)#	Alinity i HIV Ag/Ab Combo Next (S/CO)#*	RealTi*me* HIV-1 Viral Load (Log copies/mL)	Alinity m HIV-1 Viral Load (Log copies/mL)*	Determine HIV-1/2 Ag/Ab Combo RDT (p24 Ag)	Determine HIV-1/2 Ag/Ab Combo RDT (Ab)	Subtype Classification
11	PX241578	2017-04-26	366.99	63.59	5.34	5.31	-	+	A
15	PX241579	2017-05-31	36.75	32.14	4.95	5.00	-	+	D
16	PX241580	2017-06-09	205.47	48.57	5.90	5.94	-	+	D
21	PX241581	2017-07-19	891.84	N/A	5.75	N/A	N/A	N/A	URF
24	PX241582	2017-03-15	860.16	264.81	5.85	5.94	-	+	F1
27	PX241583	2017-04-05	850.52	314.96	5.80	5.96	-	+	URF
33	PX241584	2017-05-10	1051.18	369.68	5.48	5.89	-	+	CRF02_AG
35	PX241585	2017-05-10	569.5	N/A	6.26	N/A	N/A	N/A	H
37	PX241586	2017-05-10	615.27	N/A	5.85	N/A	N/A	N/A	G
38	PX241587	2017-05-19	988.87	400.3	5.51	5.54	-	+	C
40	PX241588	2017-05-19	992.65	447.51	5.24	5.60	-	+	A
42	PX241589	2017-05-19	664.12	266.25	5.78	5.66	-	+	A
44	PX241590	2017-05-24	897.91	348.92	5.15	5.25	-	+	A
48	PX241591	2017-06-07	1164.29	458.21	5.24	5.22	-	+	A
49	PX241592	2017-06-15	710.81	N/A	5.12	N/A	N/A	N/A	H
51	PX241593	2017-06-15	611.62	262.61	6.79	6.93	-	+	A
53	PX241594	2017-07-05	562.94	N/A	4.89	N/A	N/A	N/A	G
55	PX241595	2017-05-10	1107.66	N/A	4.87	N/A	N/A	N/A	H
56	PX241596	2017-05-11	27.35	N/A	5.05	N/A	N/A	N/A	G
57	PX241597	2017-05-11	705.38	228.12	5.43	5.55	-	+	G
62	PX241598	2017-06-06	755.92	212.91	5.09	5.39	-	+	D
65	PX241599	2017-03-14	951.04	420.33	5.02	5.21	-	+	CRF02_AG
70	PX241600	2017-05-19	255.57	61.77	5.78	5.71	-	+	CRF45_cpx
78	PX241601	2017-07-17	637.07	222.3	5.54	5.62	-	+	C

Specimen Identifier	Genbank Accession Number	Collection date	ARCHITECT HIV Ag/Ab Combo (S/CO)#	Alinity i HIV Ag/Ab Combo Next (S/CO)#*	RealTime HIV-1 Viral Load (Log copies/mL)	Alinity m HIV-1 Viral Load (Log copies/mL)*	Determine HIV-1/2 Ag/Ab Combo RDT (p24 Ag)	Determine HIV-1/2 Ag/Ab Combo RDT (Ab)	Subtype Classification
81	PX241602	2017-04-21	268.62	60.55	5.38	5.50	-	+	A
82	PX241603	2017-04-21	790.74	214.43	5.25	5.41	-	+	URF
91	PX241604	2017-03-09	955.14	391.87	6.04	5.86	-	+	A
92	PX241605	2017-03-09	973.59	N/A	5.76	N/A	N/A	N/A	URF
96	PX241606	2017-03-15	588.3	132.69	5.55	5.57	-	+	A
99	PX241607	2017-03-23	731.69	181.03	6.22	6.00	-	+	A
100	PX241608	2017-03-23	883.92	N/A	5.72	N/A	N/A	N/A	URF
101	PX241609	2017-03-23	1007.34	411.23	4.61	4.56	-	+	CRF25_cpx
102	PX241610	2017-03-23	580.62	156.02	5.81	5.93	-	+	A
103	PX241611	2017-03-23	799.95	245.41	4.99	4.79	-	+	URF
106	PX241612	2017-04-28	46.18	51.04	4.93	4.64	-	+	A
108	PX241613	2017-04-28	566.87	181.81	5.33	5.22	-	+	URF
109	PX241614	2017-04-28	79.2	48.59	6.58	6.46	-	+	URF
110	PX241615	2017-04-28	651.93	214.56	5.48	5.71	-	+	A
112	PX241616	2017-05-08	147.55	51.6	5.06	5.01	-	+	D
117	PX241617	2017-05-08	522.38	274.69	5.39	5.53	-	+	URF
125	PX241618	2017-07-17	629.72	265.67	5.78	5.71	-	+	CRF01_AE
126	PX241619	2017-08-03	618.77	296.25	5.46	5.62	-	+	C
131	PX241620	2017-07-24	127.6	30.45	6.94	6.85	-	+	URF
132	PX241621	2017-07-24	550.27	192.58	6.03	6.10	-	+	URF
134	PX241622	2017-04-13	565.62	223.11	5.47	5.31	-	+	F1
136	PX241623	2017-08-03	797.68	N/A	6.06	N/A	N/A	N/A	U
142	PX241624	2017-07-18	845.97	N/A	6.15	N/A	N/A	N/A	URF
144	PX241625	2017-06-08	989.97	192.93	4.88	4.21	-	+	A
145	PX241626	2017-06-09	911.12	309.25	5.09	5.07	-	+	CRF25_cpx
146	PX241627	2017-06-26	128.63	N/A	5.80	N/A	N/A	N/A	H

Specimen Identifier	Genbank Accession Number	Collection date	ARCHITECT HIV Ag/Ab Combo (S/CO)#	Alinity i HIV Ag/Ab Combo Next (S/CO)#*	RealTime HIV-1 Viral Load (Log copies/mL)	Alinity m HIV-1 Viral Load (Log copies/mL)*	Determine HIV-1/2 Ag/Ab Combo RDT (p24 Ag)	Determine HIV-1/2 Ag/Ab Combo RDT (Ab)	Subtype Classification
150	PX241628	2017-07-18	91.73	53.09	6.06	6.63	-	+	D
151	PX241629	2017-07-26	752.91	241.18	5.59	5.33	-	+	F1
154	PX241630	2017-07-03	657.82	262.74	5.83	5.59	-	+	H
157	PX241631	2017-07-27	731.97	278.88	5.35	5.31	-	+	URF
159	PX241632	2017-06-26	549.49	95.79	5.48	5.33	-	+	C
160	PX241633	2017-07-07	745.79	N/A	5.60	N/A	N/A	N/A	URF
161	PX241634	2017-07-07	818.1	N/A	5.98	N/A	N/A	N/A	URF
166	PX241635	2017-07-26	852.13	N/A	5.13	N/A	N/A	N/A	H
169	PX241636	2017-08-03	911.09	381.82	4.68	5.38	-	+	C
179	PX241637	2017-06-01	573.24	N/A	5.04	N/A	N/A	N/A	URF
181	PX241638	2017-06-13	970.69	274.14	3.65	4.20	-	+	H
188	PX241639	2017-07-01	895.97	261.64	4.56	4.43	-	+	C
190	PX241640	2017-07-03	830.66	307.01	5.34	5.40	-	+	G
191	PX241641	2017-07-06	691.23	180.29	5.69	6.31	-	+	A
193	PX241642	2017-07-15	605.45	274.19	5.77	5.22	-	+	CRF02_AG
196	PX241643	2017-05-12	810.38	N/A	3.42	N/A	N/A	N/A	C
205	PX241644	2017-07-04	935.69	314	5.62	5.59	-	+	CRF45_cpx
206	PX241645	2017-07-04	1027.46	374.45	5.48	5.33	-	+	URF
208	PX241646	2017-07-18	817.72	N/A	4.79	N/A	N/A	N/A	C
216	PX241647	2017-07-27	299.56	75.73	5.05	5.24	-	+	URF
218	PX241648	2017-03-09	517.01	166.88	5.57	5.37	-	+	CRF45_cpx
220	PX241649	2017-07-18	17.65	30.04	5.75	5.86	-	+	C
222	PX241650	2017-07-25	498.11	160.39	4.99	5.00	-	+	CRF45_cpx
224	PX241651	2017-08-03	1053.96	326.54	5.92	6.11	-	+	U
231	PX241652	2017-06-12	553.36	137	5.96	6.12	-	+	F1
237	PX241653	2017-06-13	1051.59	N/A	4.20	N/A	N/A	N/A	C

Specimen Identifier	Genbank Accession Number	Collection date	ARCHITECT HIV Ag/Ab Combo (S/CO)#	Alinity i HIV Ag/Ab Combo Next (S/CO)#*	RealTime HIV-1 Viral Load (Log copies/mL)	Alinity m HIV-1 Viral Load (Log copies/mL)*	Determine HIV-1/2 Ag/Ab Combo RDT (p24 Ag)	Determine HIV-1/2 Ag/Ab Combo RDT (Ab)	Subtype Classification
242	PX241654	2017-06-13	771.12	N/A	3.41	N/A	N/A	N/A	H
247	PX241655	2017-06-28	184.32	49.62	4.76	4.86	-	+	D
252	PX241656	2017-06-28	174.59	61.4	5.81	5.78	-	+	CRF02_AG
257	PX241657	2017-05-24	732.12	N/A	5.41	N/A	N/A	N/A	A
263	PX241658	2017-05-26	722.07	N/A	5.11	N/A	N/A	N/A	C
264	PX241659	2017-05-26	1038	N/A	5.33	N/A	N/A	N/A	C
266	PX241660	2017-05-26	177.49	N/A	6.06	N/A	N/A	N/A	C
406	PX241661	2017-04-20	827.33	324.35	4.41	4.67	-	+	CRF11_cpx
416	PX241662	2017-04-21	571.11	226.19	4.15	4.08	-	+	C
482	PX241663	2017-07-19	978.26	204.76	8.13	7.60	-	+	G
519	PX241664	2017-04-26	233.14	59.14	5.61	5.71	-	+	G
569	PX241665	2017-05-31	830.88	296.83	6.91	7.00	-	+	G
629	PX241666	2017-07-12	1048.23	N/A	4.37	N/A	N/A	N/A	K
1230	PX241667	2017-07-22	1022.39	480.36	5.96	5.93	-	+	URF
1902	PX241668	2017-07-05	985.5	N/A	5.60	N/A	N/A	N/A	CRF92_C2U
18CD-0003	PX241669	2017-09-13	694.42	217.62	4.74	5.04	-	+	D
18CD-0019	PX241670	2017-10-26	499.38	138.82	4.33	4.41	-	+	URF
18CD-0026	PX241671	2017-11-06	675.18	218.42	5.24	5.62	-	+	CRF11_cpx
18CD-0041	PX241672	2017-12-06	1002.19	N/A	5.52	N/A	N/A	N/A	A
18CD-0056	PX241673	2018-01-03	615.97	198.38	5.75	5.70	-	+	URF
18CD-0068	PX241674	2018-01-22	903.94	369.4	5.21	5.21	-	+	B
18CD-0082	PX241675	2018-03-13	549.55	190.24	6.08	5.87	-	+	C
18CD-0085	PX241676	2018-03-27	969.26	416.23	4.72	5.24	-	+	URF
18CD-0087	PX241677	2018-02-13	660.54	153.96	5.49	5.53	-	+	C
18CD-0097	PX241678	2018-03-14	692.57	203.85	4.68	4.93	-	+	URF
18CD-0098	PX241679	2018-03-15	751.21	295.07	4.56	4.85	-	+	URF

Specimen Identifier	Genbank Accession Number	Collection date	ARCHITECT HIV Ag/Ab Combo (S/CO)#	Alinity i HIV Ag/Ab Combo Next (S/CO)#*	RealTi*me* HIV-1 Viral Load (Log copies/mL)	Alinity m HIV-1 Viral Load (Log copies/mL)*	Determine HIV-1/2 Ag/Ab Combo RDT (p24 Ag)	Determine HIV-1/2 Ag/Ab Combo RDT (Ab)	Subtype Classification
18CD-0102	PX241680	2018-03-30	613.82	144.24	4.96	4.90	-	+	CRF01_AE
18CD-0105	PX241681	2017-08-10	455.24	172.21	5.57	5.04	-	+	CRF13_cpx
18CD-0112	PX241682	2017-09-13	704.02	211.25	5.09	4.99	-	+	C
18CD-0113	PX241683	2017-09-18	416.42	125.83	5.18	5.04	-	+	URF
18CD-0114	PX241684	2017-10-03	588.2	243.37	5.84	5.92	-	+	URF
18CD-0116	PX241685	2017-10-05	592.35	149.01	4.46	4.62	-	+	U
18CD-0117	PX241686	2017-10-13	976.85	452.65	5.69	5.85	-	+	URF
18CD-0127	PX241687	2017-11-21	961.48	465.94	4.38	4.45	-	+	G
18CD-0139	PX241688	2018-01-02	410.47	175.56	4.23	4.24	-	+	URF
18CD-0144	PX241689	2018-01-26	385.12	76.41	5.62	5.74	-	+	A
18CD-0145	PX241690	2018-01-31	607.94	158.76	5.42	5.72	-	+	A
18CD-0148	PX241691	2018-02-02	780.07	217.22	5.64	5.56	-	+	A
18CD-0152	PX241692	2017-11-17	818.75	267.62	5.49	5.05	-	+	CRF45_cpx
18CD-0161	PX241693	2017-12-21	826.54	202.72	3.35	3.63	-	+	G
18CD-0165	PX241694	2018-03-20	545.05	166.35	5.27	5.07	-	+	URF
18CD-0217	PX241695	2018-02-05	206.12	56.48	3.89	4.65	-	+	F1
18CD-0241	PX241696	2017-08-12	513.65	169.19	6.12	5.88	-	+	A
18CD-0242	PX241697	2017-08-12	496	171.9	6.07	5.85	-	+	A
18CD-0251	PX241698	2017-09-13	784.89	275.5	5.01	5.18	-	+	A
18CD-0252	PX241699	2017-09-13	812.23	261.97	5.02	4.81	-	+	A
18CD-0257	PX241700	2018-02-26	963.91	467.57	5.62	5.86	-	+	C
18CD-0259	PX241701	2018-02-26	783.42	328.58	5.65	5.69	-	+	J
18CD-0260	PX241702	2018-02-26	778.87	340.75	4.87	5.07	-	+	C
18CD-0268	PX241703	2018-03-18	837.02	176.23	5.47	5.82	-	+	A
18CD-0295	PX241704	2017-12-05	947.49	376.51	5.95	5.58	-	+	A
18CD-0352	PX241705	2018-02-02	894.51	248.5	5.56	5.78	-	+	C

Specimen Identifier	Genbank Accession Number	Collection date	ARCHITECT HIV Ag/Ab Combo (S/CO)#	Alinity i HIV Ag/Ab Combo Next (S/CO)#*	RealT*ime* HIV-1 Viral Load (Log copies/mL)	Alinity m HIV-1 Viral Load (Log copies/mL)*	Determine HIV-1/2 Ag/Ab Combo RDT (p24 Ag)	Determine HIV-1/2 Ag/Ab Combo RDT (Ab)	Subtype Classification
18CD-0370	PX241706	2017-09-08	565.3	171.25	6.06	5.77	-	+	URF
18CD-0380	PX241707	2017-12-27	371.21	81.6	4.37	4.52	-	+	C
18CD-0392	PX241708	2018-02-05	232.29	51.07	3.87	4.56	-	+	F1
18CD-0432	PX241709	2017-12-14	626.94	191.03	5.33	5.23	-	+	G
18CD-0456	PX241710	2017-11-10	841.03	324.96	5.71	5.70	-	+	CRF13_cpx
18CD-0468	PX241711	2017-12-27	476.58	197.76	5.10	5.22	-	+	CRF02_AG
18CD-0514	PX241712	2018-02-22	957.96	287.27	4.93	4.83	-	+	URF
18CD-0519	PX241713	2018-03-22	817	353.79	4.82	5.01	-	+	URF
18CD-0543	PX241714	2017-12-26	843.98	343.05	6.00	6.05	-	+	D
18CD-0565	PX241715	2018-02-26	403.7	224.69	4.94	5.14	-	+	CRF92_C2U
18CD-0577	PX241716	2017-09-04	993.26	495.95	5.48	5.58	-	+	CRF45_cpx
18CD-0579	PX241717	2017-09-13	367.09	101.25	5.10	5.17	-	+	G
18CD-0595	PX241718	2017-09-26	317.28	93.77	5.97	6.28	-	+	G
18CD-0597	PX241719	2017-10-02	293.44	69.77	4.90	5.12	-	+	URF
18CD-0607	PX241720	2017-11-22	221.92	60.79	5.49	5.52	-	+	CRF02_AG
18CD-0610	PX241721	2017-11-23	581.4	232.85	4.33	5.08	-	+	K/H
18CD-0648	PX241722	2018-03-01	937.18	300.31	5.83	6.26	-	+	URF
18CD-0657	PX241723	2017-09-10	544.09	155.43	5.77	5.91	-	+	URF
18CD-0662	PX241724	2018-03-14	1025.39	451.19	6.23	6.52	-	+	C
18CD-0670	PX241725	2017-09-30	589.32	345.3	4.89	5.10	-	+	F1
18CD-0675	PX241726	2017-11-17	680.36	140.67	5.70	5.89	-	+	A
18CD-0688	PX241727	2017-09-14	775.73	277.5	5.42	5.99	-	+	URF
18CD-0693	PX241728	2017-10-09	1101.71	379.12	5.24	5.62	-	+	C
18CD-0698	PX241729	2017-11-20	856.95	306.35	7.13	7.05	-	+	F1
18CD-0705	PX241730	2018-01-18	601.55	289.5	5.27	5.57	-	+	URF
18CD-0713	PX241731	2018-01-19	820.98	310.57	4.84	5.28	-	+	A

Specimen Identifier	Genbank Accession Number	Collection date	ARCHITECT HIV Ag/Ab Combo (S/CO)#	Alinity i HIV Ag/Ab Combo Next (S/CO)#*	RealTi*me* HIV-1 Viral Load (Log copies/mL)	Alinity m HIV-1 Viral Load (Log copies/mL)*	Determine HIV-1/2 Ag/Ab Combo RDT (p24 Ag)	Determine HIV-1/2 Ag/Ab Combo RDT (Ab)	Subtype Classification
18CD-0745	PX241732	2017-11-16	701.62	185.08	5.57	5.45	-	+	C
18CD-0759	PX241733	2018-01-29	758.1	298.23	5.28	5.65	-	+	C
18CD-0764	PX241734	2018-02-16	747.64	234.44	5.98	6.81	-	+	URF
18CD-0777	PX241735	2017-08-23	213.51	142.91	4.79	5.07	-	+	F1
18CD-0815	PX241736	2018-01-22	919.77	322.02	4.71	4.77	-	+	H
18CD-0853	PX241737	2017-10-13	1062.41	364.56	4.95	5.20	-	+	URF
18CD-0863	PX241738	2017-11-23	25.84	45.35	4.82	5.20	-	+	D
18CD-0871	PX241739	2018-01-15	609.66	169.44	5.97	6.18	-	+	A
18CD-2118	PX241740	2018-03-26	873.72	362.31	5.19	5.59	-	+	CRF25_cpx
18CD-2588	PX241741	2018-02-14	1049.11	264.8	7.40	7.26	-	+	URF
CD19-0017	PX241742	2018-05-19	645.01	174.82	5.25	5.23	-	+	URF
CD19-0056	PX241743	2018-08-20	770.28	224.57	4.39	3.95	-	+	G
CD19-0059	PX241744	2018-09-04	834.58	266.49	5.41	5.55	-	+	G
CD19-0070	PX241745	2019-01-09	713.82	190.59	4.87	5.19	-	+	URF
CD19-0079	PX241746	2019-02-16	659.64	N/A	6.44	N/A	N/A	N/A	F1
CD19-0214	PX241747	2018-06-25	100.19	44.43	5.95	5.80	-	+	D
CD19-0215	PX241748	2018-06-28	1154.02	433.38	5.54	5.83	-	+	A
CD19-0358	PX241749	2018-05-16	957.87	N/A	4.76	N/A	N/A	N/A	A
CD19-0396	PX241750	2018-04-27	1015	345.99	5.98	6.16	-	+	G
CD19-0418	PX241751	2018-04-10	662.03	N/A	5.46	N/A	N/A	N/A	URF
CD19-0425	PX241752	2018-04-23	597.36	N/A	4.96	N/A	N/A	N/A	C
CD19-0433	PX241753	5-2018	1007.28	N/A	5.81	N/A	N/A	N/A	C
CD19-0440	PX241754	2018-04-23	961.73	N/A	6.09	N/A	N/A	N/A	A
CD19-0443	PX241755	5-2018	79.38	48.64	6.29	6.32	-	+	D
CD19-0451	PX241756	2018-04-23	660.24	328.58	5.15	5.17	-	+	D
CD19-0478	PX241757	2018-04-02	314.19	88.44	5.50	5.69	-	+	CRF13_cpx

Specimen Identifier	Genbank Accession Number	Collection date	ARCHITECT HIV Ag/Ab Combo (S/CO)#	Alinity i HIV Ag/Ab Combo Next (S/CO)#*	RealTi*me* HIV-1 Viral Load (Log copies/mL)	Alinity m HIV-1 Viral Load (Log copies/mL)*	Determine HIV-1/2 Ag/Ab Combo RDT (p24 Ag)	Determine HIV-1/2 Ag/Ab Combo RDT (Ab)	Subtype Classification
CD19-0492	PX241758	5-2018	1140.48	470.03	5.53	5.49	-	+	G
CD19-0538	PX241759	5-2018	622.2	226.17	4.83	5.26	-	+	G
CD19-0540	PX241760	2018-04-07	1080.61	N/A	3.73	N/A	N/A	N/A	URF
CD19-0579	PX241761	2018-04-30	1068.62	374.79	5.70	5.91	-	+	D
CD19-0588	PX241762	2018-04-28	246.7	77.8	5.92	5.55	-	+	CRF45_cpx
CD19-0605	PX241763	2018-04-17	525.5	165.24	5.54	5.64	-	+	CRF01_AE
CD19-0606	PX241764	2018-04-17	404.48	93.38	4.77	4.74	-	+	URF
CD19-0608	PX241765	2018-04-25	559.6	215.27	5.85	5.45	-	+	CRF45_cpx
CD19-0625	PX241766	5-2018	541.15	219.1	5.90	6.19	-	+	A
CD19-0660	PX241767	5-2018	900	357.53	5.79	5.83	-	+	CRF25_cpx
CD19-0661	PX241768	5-2018	610.83	296.39	6.15	6.40	-	+	URF
CD19-0695	PX241769	5-2018	726.8	283.34	5.84	5.30	-	+	URF
CD19-0700	PX241770	2018-05-09	451.3	97.26	5.73	5.91	-	+	F1
CD19-0719	PX241771	5-2018	287.82	N/A	4.99	N/A	N/A	N/A	URF
CD19-0731	PX241772	5-2018	443.44	124.44	6.34	6.18	-	+	CRF05_DF
CD19-0734	PX241773	5-2018	750.45	N/A	6.85	N/A	N/A	N/A	URF
CD19-0739	PX241774	2018-04-10	100.17	64.69	5.82	5.79	-	+	D

# S/CO >1.0 is Reactive *Alinity i HIV Ag/Ab Combo Next and Alinity m viral load, Tested at 1:10 dilution

Supplemental Table S2: Summary of participant demographics and study collection sites

Sex	N= (%)
Male	39/197 (19.8)
Female	77/197 (39.1)
Unknown	81/197 (41.1)
**Total**	197

Age (n=)	Median (IQR)
Male (39)	42 (35-52)
Female (76)	41 (32-47.75)
Unknown (2)	57 (45-69)
**Total**	42 (32.5-49)

Collection Site	Specimens (n=)
CENTRE DE SANTE BONDEKO	4
CENTRE DE SANTE DE LA DEUXIEME RUE	13
CENTRE DE SANTE ELIMO SANTU	2
CENTRE DE SANTE ELONGA	3
CENTRE DE SANTE ET MATERNITE LISANGA	11
CENTRE DE SANTE KINKENDA	2
CENTRE DE SANTE LISUNGI	14
CENTRE DE SANTE MOBENGI	8
CENTRE DE SANTE MOKENGELI	1
CENTRE DE SANTE MOSSOSO	1
CENTRE DE SANTE PILOTE DE MASINA	10
CENTRE DE SANTE SAINT ALPHONSE	26
CENTRE DE SANTE SAINT PIERRE	8
CENTRE HOSPITALIER ROI BAUDOUIN Ier	22
CENTRE MERE ET ENFANT DE BANDALUNGWA	1
CENTRE MERE ET ENFANT DE BARUMBU	6
CENTRE MERE ET ENFANT DE BUMBU	3
CLINIQUE BONDEKO	2
CLINIQUE ET MATERNITE OEUVRE SOCIALE POUR LE DEVELOPPEMENT	1
HOPITAL CENTRAL DE LA POLICE	1
HOPITAL DES SOEURS DE KINGASANI	2
HOPITAL D'ETAT MAMAN PAMELA	1
HOPITAL DU CAMP LUFUNGULA	3
HOPITAL GENERAL DE REFERENCE DE MAKALA	1
HOPITAL GENERAL DE REFERENCE DE VANGA	5
HOPITAL GENERAL PROVINCIAL DE REFERENCE DE KINSHASA	3
HOPITAL MILITAIRE REGIONAL CAMP KOKOLO	3
KOKOLO HOPITAL	21
MATERNITE DE BINZA	16
MATERNITE DE KINTAMBO	1
POLYCLINIQUE SAINT JOSEPH	1
Unknown	1
**Total**	197

Supplemental Table S3: HIV-1 Group M references used for phylogenetic trees presented in Fig. 1 of the main text

Subtype/CRF	Country	Year	Specimen ID	Accession number.
CPZ	GA	1988	GAB1	X52154
A1	UG	1998	98UG57136	AF484509
A1	UG	1992	92UG037-A40	AB253429
A1	KE	1994	Q23-17	AF004885
A1	SE	1994	SE7253	AF069670
A2	CY	1994	94CY017-41	AF286237
A2	CD	1997	97CDKTB48	AF286238
A2	CM	2001	01CM-1445MV	GU201516
A3	SN	2001	DDI579	AY521629
A3	SN	1996	DDJ360	AY521630
A3	SN	2001	DDJ369	AY521631
A4	CD	2002	02CD-KTB035	AM000055
A4	CD	1997	97CD-KCC2	AM000053
A4	CD	1997	97CD-KTB13	AM000054
A6	IT	2002	60000	EU861977
A6	UA	2000	98UA0116	AF413987
A6	UA	2012	DEMA112UA024	KU749403
A7	NG	2009	09NG010499	KX389622
A7	NG	2010	10NG040248	KX389608
A7	NG	2011	DEURF11NG005	MH078558
A8	CV	2010	CV-10-115	MW353966
A8	CV	2010	CV-10-126	MW353967
B	US	1998	1058-11	AY331295
B	NL	2000	671-00T36	AY423387
B	FR	1983	HXB2-LAI-IIIB-BRU	K03455
B	US	1986	JRFL-JR-FL	U63632
B	US	1983	RF-HAT3	M17451
B	TH	1990	BK132	AY173951
B	US	1990	WEAU160-GHOSH	U21135

Subtype/CRF	Country	Year	Specimen ID	Accession number.
C	BR	1992	BR025-d	U52953
C	IN	1995	95IN21068	AF067155
C	BW	1996	96BW0502	AF110967
C	ET	1986	ETH2220	U46016
C	ZA	2004	04ZASK146	AY772699
D	CM	2001	01CM-4412HAL	AY371157
D	CD	1984	84ZR085	U88822
D	UG	1994	94UG114	U88824
D	CD	1983	ELI	K03454
D	CD	1983	NDK	M27323
D	TZ	2001	A280	AY253311
F1	BR	1993	93BR020-1	AF005494
F1	FI	1993	FIN9363	AF075703
F1	FR	1996	96FR-MP411	AJ249238
F1	BE	1993	VI850	AF077336
F2	CM	2002	02CM-0016BBY	AY371158
F2	CM	1997	CM53657	AF377956
F2	CM	1995	95CM-MP255	AJ249236
F2	CM	1995	95CM-MP257	AJ249237
G	NG	1992	92NG083-JV10832	U88826
G	BE	1996	DRCBL	AF084936
G	KE	1993	HH8793-12-1	AF061641
G	SE	1993	SE6165-G6165	AF061642
H	CF	1990	56	AF005496
H	BE	1993	VI991	AF190127
H	BE	1993	VI997	AF190128
H	GB	2000	00GBAC4001	FJ711703
J	SE	1994	SE9173-7022	AF082395
J	SE	1993	SE9280-7887	AF082394
J	CD	1997	J-97DC-KTB147	EF614151
J	CM	2004	04CMU11421	GU237072
K	CD	1997	97ZR-EQTB11	AJ249235
K	CM	1996	96CM-MP535	AJ249239
K	CD	1987	P3844	MH705156
L	CD	1983	83CD003-Z3	AF286236
L	CD	1990	90CD121E12	AF457101
L	CD	2001	L-CG-0018a-01	MN271384
U	GR	1999	99GR303	AY046058
CRF01-AE	CF	1990	90CF11697	AF197340
CRF01-AE	CF	1990	90CR402-CAR-E-4002	U51188
CRF01-AE	CF	1990	90CF4071	AF197341
CRF01-AE	TH	1990	CM240	U54771

Subtype/CRF	Country	Year	Specimen ID	Accession number.
CRF01-AE	AF	2007	569M	GQ477441
CRF01-AE	CN	2005	05GX001	GU564221
CRF02-AG	CM	1997	97CM-MP807	AJ286133
CRF02-AG	FR	1991	DJ264	AF063224
CRF02-AG	NG	N/A	IBNG	L39106
CRF02-AG	SE	1994	SE7812	AF107770
CRF03-A6B	BY	2000	98BY10443	AF414006
CRF03-A6B	RU	1997	KAL153-2	AF193276
CRF03-A6B	RU	1998	RU98001-98RU001	AF193277
CRF04-cpx	GR	1991	GR11-97PVCH	AF119820
CRF04-cpx	GR	1997	GR84-97PVMY	AF119819
CRF04-cpx	CY	1994	94CY032-3	AF049337
CRF05-DF	ES	1999	X492	AY227107
CRF05-DF	BE	1993	VI961	AF076998
CRF05-DF	BG	2009	V-09-003	MH746260
CRF06-cpx	ML	1995	95ML127	AJ288982
CRF06-cpx	ML	1995	95ML84	AJ245481
CRF06-cpx	SN	1997	97SE1078	AJ288981
CRF06-cpx	AU	1996	BFP90	AF064699
CRF07-BC	CN	1997	97CN001-C54	AF286226
CRF07-BC	CN	1998	98CN009	AF286230
CRF07-BC	CN	2005	XJDC6431-2	EF368372
CRF08-BC	CN	1998	98CN006	AF286229
CRF08-BC	CN	1997	97CNGX-6F	AY008715
CRF08-BC	CN	1997	97CNGX-7F	AY008716
CRF09-cpx	SN	1995	95SN1795	AY093603
CRF09-cpx	SN	1995	95SN7808	AY093604
CRF09-cpx	GH	1996	96GH2911	AY093605
CRF09-cpx	US	1999	99DE4057	AY093607
CRF10-CD	TZ	1996	96TZ-BF061	AF289548
CRF10-CD	TZ	1996	96TZ-BF071	AF289549
CRF10-CD	TZ	1996	96TZ-BF110	AF289550
CRF11-cpx	CM	1995	95CM-1816	AF492624
CRF11-cpx	CM	1996	96CM-4496	AF492623
CRF11-cpx	CM	1997	MP818	AJ291718
CRF12-BF	AR	1997	A32879	AF408629
CRF12-BF	AR	1999	ARMA159	AF385936
CRF12-BF	UY	1999	URTR35	AF385935
CRF13-cpx	CM	2002	02CM-3226MN	AY371154
CRF13-cpx	CM	1996	96CM-1849	AF460972
CRF13-cpx	CM	1996	96CM-4164	AF460974
CRF14-BG	ES	2000	X475	AF423758

Subtype/CRF	Country	Year	Specimen ID	Accession number.
CRF14-BG	ES	2000	X605	AF450096
CRF14-BG	ES	2000	X623	AF450097
CRF15-01B	TH	1999	99TH-MU2079	AF516184
CRF15-01B	TH	2002	02TH-OUR1332	AF529573
CRF15-01B	TH	1999	99TH-R2399	AF530576
CRF16-A2D	KR	1997	97KR004	AF286239
CRF16-A2D	KE	1991	KNH1271	AY945736
CRF16-A2D	KE	2005	05KE493170V5	KT022403
CRF17-BF1	AR	1999	ARMA038	AY037281
CRF17-BF1	PY	2002	PY02-PSP0073	EU581824
CRF17-BF1	AR	2002	AR02-ARG1139	EU581825
CRF18-cpx	CM	1997	CM53379	AF377959
CRF18-cpx	CU	1999	CU14	AY586541
CRF18-cpx	CU	1999	CU68	AY894993
CRF19-cpx	CU	1999	CU29	AY588971
CRF19-cpx	CU	1999	CU38	AY588970
CRF19-cpx	CU	1999	CU7	AY894994
CRF20-BG	ES	1999	R77	AY586544
CRF20-BG	CU	1999	Cu103	AY586545
CRF20-BG	CU	2003	CB134	DQ020274
CRF21-A2D	KE	1991	KNH1254	AY945737
CRF21-A2D	KE	1999	KER2003	AF457051
CRF21-A2D	KE	1999	KSM4001	AF457072
CRF22-01A1	CM	2001	01CM-0001BBY	AY371159
CRF22-01A1	CM	2002	02CAMLT72	EU743963
CRF22-01A1	CM	2002	02CM-3097MN	GQ229529
CRF22-01A1	CM	2010	DE02210CM006	KP109500
CRF23-BG	CU	2003	CB118	AY900571
CRF23-BG	CU	2003	CB347	AY900572
CRF24-BG	CU	2003	CB471	AY900575
CRF24-BG	CU	2003	CB378	AY900574
CRF24-BG	CU	2003	CB619	AY900576
CRF25-cpx	CM	2006	06CM-BA-040	EU693240
CRF25-cpx	SA	2003	J11233	EU697906
CRF25-cpx	SA	2003	J11451	EU697908
CRF26-A5U	CD	1997	97CD-KTB119	FM877777
CRF26-A5U	CD	2002	02CD-KS069	FM877780
CRF26-A5U	CD	2002	02CD-MBTB047	FM877782
CRF27-cpx	FR	2004	04CD-FR-KZS	AM851091
CRF27-cpx	CD	1997	97CDKTB49	AJ404325
CRF27-cpx	CD	2002	02CD-LBR024	AM851090
CRF28-BF1	BR	1999	BREPM12313	DQ085872

Subtype/CRF	Country	Year	Specimen ID	Accession number.
CRF28-BF1	BR	1999	BREPM12609	DQ085873
CRF28-BF1	BR	1999	BREPM12817	DQ085874
CRF29-BF1	BR	1999	99UFRJ-1	AY455778
CRF29-BF1	BR	1999	BREPM11948	DQ085871
CRF29-BF1	BR	2001	BREPM16704	DQ085876
CRF30-0206	NE	2000	NE36	AJ508597
CRF30-0206	GH	2003	03GH195AG-06	AB286854
CRF30-0206	NE	1997	NE03	AJ508595
CRF31-BC	BR	2004	04BR137	AY727526
CRF31-BC	BR	2004	04BR142	AY727527
CRF31-BC	BR	2002	110PA	EF091932
CRF32-06A6	EE	2001	EE0359	AY535659
CRF32-06A6	EE	2001	EE0369	AY535660
CRF32-06A6	EE	2002	EST2002-1169	DQ167215
CRF33-01B	MY	2005	05MYKL007-1	DQ366659
CRF33-01B	MY	2005	05MYKL015-2	DQ366660
CRF33-01B	MY	2005	05MYKL045-1	DQ366662
CRF34-01B	TH	1999	OUR1969P	EF165539
CRF34-01B	TH	1999	OUR2275P	EF165540
CRF34-01B	TH	1999	OUR2478P	EF165541
CRF35-A1D	AF	2005	05AF026	EF158043
CRF35-A1D	AF	2005	05AF094	EF158040
CRF35-A1D	AF	2005	05AF095	EF158041
CRF36-cpx	CM	2007	BS40	KR017774
CRF36-cpx	CM	2000	00CMNYU1162	EF087995
CRF36-cpx	CM	2000	00CMNYU830	EF087994
CRF37-cpx	CM	1997	CM53392	AF377957
CRF37-cpx	CM	2006	1130-39	KP718917
CRF37-cpx	CM	2000	00CMNYU926	EF116594
CRF38-BF1	UY	2003	UY03-3389	FJ213783
CRF38-BF1	UY	2004	UY04-3987	FJ213781
CRF38-BF1	UY	2005	UY05-4752	FJ213780
CRF39-BF1	BR	2003	03BRRJ103	EU735534
CRF39-BF1	BR	2003	03BRRJ327	EU735536
CRF39-BF1	BR	2004	04BRRJ179	EU735535
CRF40-BF1	BR	2004	04BRSQ46	EU735540
CRF40-BF1	BR	2004	04BRRJ115	EU735538
CRF40-BF1	BR	2005	05BRRJ200	EU735539
CRF41-CD	TZ	2005	CO6577V5	KX907411
CRF41-CD	TZ	2003	CO6650V1	KX907417
CRF41-CD	TZ	2003	CO6952V1	KX907430
CRF42-BF1	LU	2005	luBF-13-05	EU170136

Subtype/CRF	Country	Year	Specimen ID	Accession number.
CRF42-BF1	LU	2003	luBF-09-03	EU170150
CRF42-BF1	LU	2004	luBF-12-04	EU170142
CRF43-02G	SA	2003	J11223	EU697904
CRF43-02G	SA	2003	J11243	EU697907
CRF43-02G	SA	2003	J11456	EU697909
CRF44-BF1	CL	2001	CH12	AY536235
CRF44-BF1	CL	2000	CH80	FJ358521
CRF44-BF1	PE	2016	DEURF16PE007	MH078557
CRF45-cpx	CD	1997	97CD-MBFE185	FN392874
CRF45-cpx	CM	1997	97CM-MP814	FN392876
CRF45-cpx	GA	1997	97GA-TB45	FN392877
CRF46-BF1	BR	2001	01BR087	DQ358801
CRF46-BF1	BR	2007	07BR-FPS625	HM026456
CRF46-BF1	BR	2007	07BR-FPS742	HM026457
CRF47-BF1	ES	2008	X2457-2	FJ670529
CRF47-BF1	ES	2008	P1942	GQ372987
CRF47-BF1	BR	2010	10BR-RJ026	KJ849798
CRF48-01B	MY	2007	07MYKT014	GQ175881
CRF48-01B	MY	2007	07MYKT016	GQ175882
CRF48-01B	MY	2007	07MYKT021	GQ175883
CRF49-cpx	GM	2002	N18380	HQ385477
CRF49-cpx	GM	1997	N28353	HQ385478
CRF49-cpx	GM	2003	N26677	HQ385479
CRF50-A1D	GB	2000	8179	JN417236
CRF50-A1D	GB	2010	12792	JN417240
CRF50-A1D	GB	2010	11762	JN417241
CRF51-01B	SG	2011	11SG-HM021	JN029801
CRF51-01B	SG	2011	11SG-HM091	JN029803
CRF51-01B	MN	2009	08MNG4608	LC312713
CRF52-01B	TH	2000	00TH-R1741	AY945734
CRF52-01B	TH	1996	M043	DQ354113
CRF52-01B	MY	2003	03MYKL018-1	DQ366664
CRF53-01B	MY	2004	04MYKL016-1	DQ366663
CRF53-01B	MY	2011	11FIR164	JX390610
CRF53-01B	MY	2010	10MYKJ079	JX390611
CRF54-01B	MY	2007	07MYKLD49	EU031915
CRF54-01B	MY	2009	09MYSB023	JX390976
CRF54-01B	MY	2008	08MYKL044	JX390977
CRF55-01B	CN	2010	HNCS102056	JX574661
CRF55-01B	CN	2011	GDDG318	JX574662
CRF55-01B	CN	2011	GDDG095	JX574663
CRF56-cpx	FR	2010	URF5-patient-A	JN882655

Subtype/CRF	Country	Year	Specimen ID	Accession number.
CRF56-cpx	FR	2010	patient-B	KC852172
CRF56-cpx	FR	2011	patient-D	KC852173
CRF56-cpx	FR	2010	patient-C	KC852174
CRF57-BC	CN	2007	341	HM776939
CRF57-BC	CN	2009	1439	JX679207
CRF57-BC	CN	2009	YNFL37	KC870044
CRF58-01B	MY	2009	09MYPR37	KC522031
CRF58-01B	MY	2011	11MY1ZK731	KC522032
CRF58-01B	MY	2011	11MY1EP794	KC522034
CRF59-01B	CN	2009	09LNA423	JX960635
CRF59-01B	CN	2011	11LNSY300392	KC462190
CRF59-01B	CN	2010	10LNSY300533	KC462191
CRF60-BC	IT	2011	BAV499	KC899079
CRF60-BC	IT	2011	BAV514	KC899080
CRF60-BC	IT	2011	BAV636	KC899081
CRF61-BC	CN	2010	JL100010	KC990124
CRF61-BC	CN	2007	FJ070004	KC990125
CRF61-BC	CN	2007	JL070009	KC990126
CRF62-BC	CN	2010	YNFL13	KC870034
CRF62-BC	CN	2010	YNFL15	KC870035
CRF62-BC	CN	2010	YNFL18	KC870037
CRF63-02A6	RU	2010	10RU6637	JN230353
CRF63-02A6	RU	2009	09RU4829	JX500701
CRF63-02A6	RU	2012	12RU15r	JX500705
CRF64-BC	CN	2010	YNFL10-1	KC870032
CRF64-BC	CN	2010	YNFL16	KC870036
CRF64-BC	CN	2009	YNFL31	KC870042
CRF65-cpx	CN	2010	YNFL01	KC870027
CRF65-cpx	CN	2010	YNFL02	KC870028
CRF65-cpx	CN	2009	YNFL05	KC870030
CRF66-BF1	ES	2017	X4352-2	MK298150
CRF66-BF1	PY	2002	02PY-PSP0093	JN251902
CRF66-BF1	PY	2002	02PY-PSP0094	JN251903
CRF67-01B	CN	2011	ANHUI-HF115	KC183779
CRF67-01B	CN	2011	ANHUI-MAS59	KC183780
CRF68-01B	CN	2011	ANHUI-WH73	KC183782
CRF68-01B	CN	2011	ANHUI-XC46	KC183783
CRF68-01B	CN	2010	JS2010001	KF758551
CRF69-01B	JP	2003	03JP-5091K231	AB845344
CRF69-01B	JP	2010	10JP-5091N200	AB845349
CRF69-01B	JP	2005	05JPMYC113SP420	LC027100
CRF70-BF1	BR	2010	10BR-PE004	KJ849758

Subtype/CRF	Country	Year	Specimen ID	Accession number.
CRF70-BF1	BR	2010	DE07010BR033	KU749388
CRF70-BF1	BR	2010	10BR-PE087	KJ849775
CRF71-BF1	BR	2010	10BR-PE084	KJ849773
CRF71-BF1	BR	2010	10BR-PE016	KJ849761
CRF71-BF1	BR	2002	02BR033	DQ358811
CRF72-BF1	BR	1999	99UFRJ-2	AY455780
CRF72-BF1	BR	2010	10BR-MG004	KJ671535
CRF72-BF1	BR	2010	10BR-MG008	KJ671536
CRF73-BG	DE	2001	9196-01	AY882421
CRF73-BG	ES	2011	X3208	KM248765
CRF74-01B	MY	2010	10MYKJ052	KR019770
CRF74-01B	MY	2010	10MYPR268	KR019771
CRF74-01B	MY	2011	11MYPR416	KR019772
CRF75-BF1	IT	2001	53143	GU595149
CRF75-BF1	IT	2002	58736	GU595160
CRF76-01B	JP	2011	D8796	MN187300
CRF76-01B	JP	2012	N628	MN187303
CRF76-01B	JP	2013	D9547	MN187302
CRF77-cpx	MY	2013	13MYNBB108	KX673818
CRF77-cpx	MY	2014	14MYNBB084	KX673819
CRF77-cpx	MY	2014	14MYNBB090	KX673820
CRF78-cpx	CN	2013	YNTC19	KU161143
CRF78-cpx	CN	2013	YNTC35	KU161144
CRF78-cpx	CN	2013	YNTC88	KU161145
CRF79-0107	CN	2015	SX15DT013	KY216146
CRF79-0107	CN	2015	SX15JC06	KY216147
CRF79-0107	CN	2015	SX15JC12	KY216148
CRF80-0107	CN	2011	YA285	MH843712
CRF80-0107	CN	2012	YA376	MH843713
CRF81-cpx	BR	2007	BRGO3001	MH986016
CRF81-cpx	BR	2008	BRMT2526	MH986017
CRF82-cpx	MM	2013	mSSDU12	KU820825
CRF82-cpx	MM	2013	mSSDU160	KU820831
CRF82-cpx	MM	2013	mSSDU75	KU820845
CRF83-cpx	MM	2013	mSSDU180	KU820834
CRF83-cpx	MM	2013	mSSDU24	KU820842
CRF83-cpx	MM	2013	mSSDU28	KU820843
CRF84-A1D	SE	2010	042SE	MF373153
CRF84-A1D	SE	2013	088SE	MF373190
CRF84-A1D	SE	2014	094SE	MF373196
CRF85-BC	CN	2014	14CN-SCYB1	KU992928
CRF85-BC	CN	2014	14CN-SCYB2	KU992929

Subtype/CRF	Country	Year	Specimen ID	Accession number.
CRF85-BC	CN	2014	14CN-SCYB20	KU992930
CRF86-BC	CN	2013	15YNHS18	KX582249
CRF86-BC	CN	2013	15YNHS23	KX582250
CRF86-BC	CN	2013	15YNHS26	KX582251
CRF87-cpx	CN	2009	09YNLC497sg	KC898992
CRF87-cpx	CN	2009	09YNRL215050sg	KC899012
CRF87-cpx	CN	2012	DH32	KF250408
CRF88-BC	CN	2005	05YNRL07sg	KC898975
CRF88-BC	CN	2005	05YNRL25sg	KC898979
CRF88-BC	CN	2009	DH19	KF250402
CRF89-BF1	BO	1999	BOL0137	AY037271
CRF89-BF1	ES	2010	P2633	KX818199
CRF89-BF1	ES	2012	P3177	KX818200
CRF90-BF1	BR	2007	BRGO3145	KY628218
CRF90-BF1	BR	2011	BRGO6043	KY628221
CRF90-BF1	BR	2010	BRGOAP801	KY628223
CRF92-C2U	CD	2008	DRC796	MF372645
CRF92-C2U	CD	2008	DRC699	MF372647
CRF92-C2U	CD	2012	VIR90	MF372650
CRF93-cpx	CD	2008	DRC367	MF372646
CRF93-cpx	CD	2008	DRC817	MF372649
CRF93-cpx	CD	2008	DRC653	MF372651
CRF94-cpx	FR	2014	24FR0113-MW71FOBV	MH141493
CRF94-cpx	FR	2015	28FR1015-MW81FOBV	MH683550
CRF94-cpx	FR	2016	05FR0916-MW95FOBV	MH141491
CRF94-cpx	FR	2016	32FR0916-MW94FOBV	MH141494
CRF95-02B	NG	2014	20168v01	MH666157
CRF95-02B	NG	2015	20268v01	MH666156
CRF95-02B	NG	2015	20428v01	MH666155
CRF96-cpx	CN	2010	JL-RF01	KF850149
CRF96-cpx	CN	2013	13YNBS54IDU	MG518476
CRF96-cpx	CN	2013	13YNBS66IDU	MG518477
CRF97-01B	LA	2011	VI29M2888	MG760399
CRF97-01B	LA	2012	BL50F5250	MG760379
CRF97-01B	LA	2012	KH18F5154	MG760380
CRF97-01B	LA	2012	VI20F5274	MG760386
CRF98-06B	FR	2009	A-Bordeaux	MH479275
CRF98-06B	FR	2014	C-Bordeaux	MH479276
CRF99-BF1	BR	2008	BRGO4028	MH986014
CRF99-BF1	BR	2009	BRGO4056	MH986013
CRF100-01C	CN	2013	YNLC27	MH909568
CRF100-01C	CN	2013	YNLC28	MH909569

Subtype/CRF	Country	Year	Specimen ID	Accession number.
CRF100-01C	CN	2013	YNLC30	MH909570
CRF101-01B	CN	2007	07CNYN370	KF835546
CRF101-01B	CN	2013	YNKM250	MK158946
CRF101-01B	CN	2013	YNZT036	MK158945
CRF102-0107	CN	2017	FY058	MN178644
CRF102-0107	CN	2018	FY336	MN178645
CRF103-01B	CN	2018	HE18S0290	MN067222
CRF103-01B	CN	2018	HE18S0298	MN067223
CRF103-01B	CN	2018	HE18S0322	MN067224
CRF104-0107	CN	2015	M62	MH396608
CRF104-0107	CN	2015	SN139	MH396609
CRF104-0107	CN	2018	MSM20183420	MK564326
CRF105-0108	CN	2014	XC2014EU01	KX353919
CRF105-0108	CN	2018	LS18S0082	MN752127
CRF105-0108	CN	2018	LS18S0127	MN752128
CRF106-cpx	CN	2015	YN15099	MT277001
CRF106-cpx	CN	2015	YN15234	MT277002
CRF106-cpx	CN	2017	17YNS377	MT276998
CRF106-cpx	CN	2018	LC18S083	MT276999
CRF107-01B	CN	2018	HL18S17	MT712388
CRF107-01B	CN	2018	HL18S214	MT712389
CRF107-01B	CN	2018	HL18S244	MT712390
CRF108-BC	ES	2016	P4439	MN172222
CRF108-BC	ES	2017	P4697	MT559132
CRF108-BC	ES	2018	P4977	MN172223
CRF108-BC	ES	2018	P5007	MN172224
CRF109-0107	CN	2014	LS11584	MT919517
CRF109-0107	CN	2014	LS14250	MT919518
CRF110-BC	CN	2007	07CNYN338	KF835524
CRF110-BC	CN	2009	09YNLC216031sg	KC898987
CRF110-BC	CN	2012	YN10189F	MW419275
CRF111-01C	CN	2016	16YN29	MT624751
CRF111-01C	CN	2016	16YN604	MT624752
CRF111-01C	CN	2016	YN16H12	MZ327297
CRF111-01C	CN	2016	YN16H18	MZ327298
CRF112-01B	CN	2018	18110456	MW018130
CRF112-01B	CN	2018	BL3766-00	MW018133
CRF112-01B	CN	2018	BL3797-00	MW018134
CRF112-01B	CN	2019	BL4450-00	MW018137
CRF113-0107	CN	2017	BL3128-00	MW018132
CRF113-0107	CN	2019	BL3957-00	MW018135
CRF113-0107	CN	2019	BL3958-00	MW018136

Subtype/CRF	Country	Year	Specimen ID	Accession number.
CRF114-0155	CN	2018	A18003	MN654104
CRF114-0155	CN	2019	B19157	MN654108
CRF114-0155	CN	2019	VCT19012	MN654109
CRF115-01C	CN	2012	kang140-NFL	KJ778896
CRF115-01C	CN	2013	kang019a-NFL	KJ778895
CRF116-0108	CN	2014	14YN263	MT624747
CRF116-0108	CN	2014	14YN264	MT624748
CRF116-0108	CN	2016	16YN253	MT624749
CRF117-0107	CN	2017	17ZJ075	MK397789
CRF118-BC	CN	2012	DH33	KF250409
CRF118-BC	CN	2013	YN23II	MZ063027
CRF118-BC	CN	2014	YN245F	MZ063028
CRF118-BC	CN	2017	YN287-168	MZ063029
CRF119-0107	CN	2017	nj82	MT347596
CRF119-0107	CN	2017	nj70	MT347595
CRF119-0107	CN	2017	nj111	MT347589
CRF120-0107	CN	2015	MSM-LS14873	OK662597
CRF120-0107	CN	2015	HES-LS16846	OK662596
CRF120-0107	CN	2015	CN-SZ-MSM-MSM-LS15083	ON351495
CRF121-0107	CN	2011	JL-RF09	KJ184180
CRF121-0107	CN	2015	MSM-LS16178	MZ076689
CRF121-0107	CN	2016	IDU-LS17859	OK584016
CRF122-BF1	ES	2009	X2632-4	KC113006
CRF122-BF1	ES	2012	GA874035	OL982321
CRF122-BF1	ES	2018	GA486085	OL982314
CRF122-BF1	ES	2011	GA099170	OL982312
CRF123-0107	CN	2020	HB030190	OM025086
CRF123-0107	CN	2020	HB010314	OM025085
CRF123-0107	CN	2020	HB010047	OM025084
CRF124-cpx	AO	2009	ANG-44	ON962803
CRF124-cpx	AO	2009	ANG-37	ON962802
CRF125-0107	CN	2018	YNZTA177	ON054294
CRF125-0107	CN	2018	YNZTA174	ON054293
CRF125-0107	CN	2018	YNZTA178	ON054292
CRF126-0755	CN	2019	ZLQ02946	ON456390
CRF126-0755	CN	2018	ZLQ00461	ON456389
CRF126-0755	CN	2021	210012	ON456387
CRF129-56G	CY	2018	CY537	ON989289
CRF129-56G	CY	2018	CY525	ON989282
CRF129-56G	CY	2018	CY512	OP781327
CRF130-A1B	CY	2017	CY397	OP781326
CRF132-94B	FR	2021	66FR1121-MW78FOBV	ON901791

Subtype/CRF	Country	Year	Specimen ID	Accession number.
CRF132-94B	FR	2019	85FR1119-MW75FOBV	ON901788
CRF132-94B	FR	2021	60FR0321-MW95FOBV	ON901787
CRF133-A6B	RU	2020	KRS189-1	OP056077
CRF133-A6B	RU	2017	KRS132-1	OP056076
CRF133-A6B	RU	2017	KRS100	OP056073
CRF134-0107	CN	2021	21ZT301	OQ747191
CRF134-0107	CN	2021	21ZT286	OQ747190
CRF134-0107	CN	2021	21ZT276	OQ747188
CRF137-0107	CN	2021	GD5530-ZLQ05913	OP716040
CRF137-0107	CN	2019	GD5515-ZLQ01542	OP716035
CRF137-0107	CN	2018	GD5511-3067	OP716032
CRF140-0107	CN	2020	HB010063	MW728361
CRF140-0107	CN	2020	9-HB100018-FLG-6	OQ749406
CRF140-0107	CN	2021	2-HB010222-FLG-2	OQ749397
CRF140-0107	CN	2020	7-HB010104-FLG-4	OQ749394
CRF143-cpx	CN	2021	YNA146	OQ948242
CRF143-cpx	CN	2013	YNA40	OQ948241
CRF143-cpx	CN	2020	YNA288	OQ948240

Supplemental Table S4: HIV-1 Group M references used for phylogenetic trees via Bayesian Inference, presented in Fig. 5 of the main text.

Subtype/CRF	Country	Year	Strain or Isolate ID	Accession Number
A	CD	1987	P4039	MH705157
A	CH	2003	HIV_CH_BID_V3538_2003	JQ403028
A	KE	1996	BG505.M27P.ENV.T1	MW650633
A	ZA	2009	707PKE02N3	HM623589
A1	BG	2009	V_09_008	MH746258
A1	CD	2002	LA01AlPr	KU168256
A1	CD	1987	PBS6126	MH705153
A1	CY	2005	CY140	FJ388938
A1	CY	2007	CY207	JF683759
A1	CY	2007	CY209	JF683761
A1	CY	2008	CY230	JF683779
A1	FI	1991	FIN91121	AF219261
A1	IN	1999	NARI_FLS_VB15	KT152842
A1	KE	2000	00KE_KER2008	AY736809
A1	KE	2000	MSA4069	AF457080
A1	KE	2000	MSA4070	AF457081
A1	KE	2000	MSA4079	AF457086
A1	KE	2004	04KE169579V3	KT022360
A1	KE	2004	04KE649309V2	KT022367
A1	KE	2005	05KE376579V4	KT022372
A1	KE	2005	05KE607907V4	KT022374
A1	KE	2005	QH343_21M_ENV_B5	FJ866120
A1	KE	2006	06KE404877V7	KT022381
A1	KE	2006	06KE661996V6	KT022383
A1	KE	2006	06KECst_025	FJ623486
A1	KE	2007	21020_13	HM215275
A1	KE	2011	20509v01_01	MN791710
A1	KE	2011	320_KE	KU921746
A1	KE	2011	366_KE	KU921756
A1	KE	2011	405_KE	KU921780
A1	KE	2011	DEMA111KE002	KF716474
A1	KE	1986	ML170_1986	AF539405
A1	KE	1990	K89_KENYA_KE89	L22943
A1	KE	1994	MF520.W14M.HH2	KX168075
A1	KE	1994	Q842_d16	AF407162
A1	KE	1995	QB850.73p.E3	KT008652
A1	KE	1996	QB726_70M_ENV_C4	FJ866112
A1	KE	1997	ML752	AY322193
A1	KE	1998	QA413_1007M_ENV_E15	FJ396018
A1	KE	1999	99KE_KNH1135	AY736814
A1	PK	2014	PK001	KX232594

Subtype/CRF	Country	Year	Strain or Isolate ID	Accession Number
A1	PK	2015	PK021	KX232614
A1	RW	2005	R57F_175020_1	MT942776
A1	RW	2006	R72M_175027_4	MT942795
A1	RW	2007	pR463F	JX236677
A1	RW	2008	PC64_M03c001	MF565934
A1	RW	2008	R1077F_175071_P1_1	MT942842
A1	RW	2008	R3103M_175079_1	MT942889
A1	RW	2010	R3671F_175092_4	MT942931
A1	RW	2011	DEMA111RW002	KF716472
A1	RW	2016	MAS_1F_Con	MZ642267
A1	RW	2017	REM_29F_Con	MZ642260
A1	RW	1993	93RW_024	AY713406
A1	SE	2012	064GR	MF373167
A1	SE	2013	079KE	MF373182
A1	TZ	2000	216_F2_A1	HQ659604
A1	TZ	2001	A341	AY253314
A1	TZ	2003	CO0543V2	KX907348
A1	TZ	2005	CO6592V5	KX907412
A1	TZ	2006	CO6830V7	KX907423
A1	TZ	2006	CO6974V7	KX907431
A1	TZ	2008	707010095_D8	HQ615968
A1	TZ	2008	CH0219_e4	HM204621
A1	TZ	2018	AKC_NV_97.NV97.1	MZ147135
A1	TZ	1997	97TZ02	AF361872
A1	UG	2009	DEMA109UG001	KF716478
A1	UG	2009	DEMA109UG017	KP109490
A1	UG	2011	10203v01_01	MN791169
A1	UG	2011	DEMA110UG009	KF716486
A1	UG	1992	UG029	AY494973
A1	UG	1998	120Flc01	EU852958
A1	US	2019	UNC_458_PL2_M19	MW062965
A1	ZA	2004	503_15344_T10_A1	KT183312
A2	CD	1987	PBS1195	MH705163
A2	CD	1997	97CDKS10	AF286241
A2	CM	2001	01CM_1445MV	GU201516
A2	CY	1994	94CY017_41	AF286237
B	AR	2009	DEMB09AR010	MH078530
B	AR	1999	ARMA132	AY037282
B	AU	1987	MBC925	AF042101
B	AU	1993	MBC18_MBCC18	AF042102
B	AU	1995	C24	AF538304
B	BE	2016	BR	MN486014
B	BE	2016	STAR09_TTM_26	MZ041647

Subtype/CRF	Country	Year	Strain or Isolate ID	Accession Number
B	BR	2003	BREPM1038	EF637048
B	BR	2004	04BR1049	JN692451
B	BR	2005	05BR1101	JN692473
B	BR	2006	06BR1115	JN692479
B	BR	2010	10BR_PE030	KT427733
B	BR	2010	10BR_PE055	KT427847
B	BR	2010	10BR_PE072	KT427709
B	BR	2010	10BR_PE082	KT427703
B	BR	2010	10BR_RJ100	KT427754
B	BR	2010	10BR_SP073_2	KT427667
B	BR	2012	2012BRRJNEUT17	KX181905
B	CA	2003	PSL024B10	GU562272
B	CA	2016	OM5162_3b_B7_ORF	OK011978
B	CA	1997	ACTDM580208A15	GU562033
B	CH	2002	T1SHCS901cl_D2_T1	KX792621
B	CH	2004	HIV_CH_BID_V4408_2004	JQ403042
B	CN	2004	CNHLJBF04023	EU131797
B	CN	2005	05CNHB_hp3	DQ990880
B	CN	2005	CNE57	HM215420
B	CN	2006	CBJC515_20060418_14	MF591612
B	CN	2008	BJOX041000.e10	KM218168
B	CN	2008	cbjc468	JF932498
B	CN	2012	134_0_27	KX692916
B	CN	2012	2039_2_13	KX693556
B	CN	2012	2362_2_19	KX693770
B	CN	1999	plwj11_6	GU647196
B	CU	2014	14CU005	KR914676
B	CY	2005	CY056	FJ388905
B	CY	2005	CY074	FJ388915
B	CY	2005	CY131	FJ388935
B	CY	2006	CY180	FJ388955
B	CY	2008	CY226	JF683775
B	CY	2009	CY254	JF683797
B	DE	2010	464704	KT124772
B	DE	1986	HAN	U43141
B	ES	2014	ARP1206	KT276266
B	ES	1989	U61	DQ854716
B	FR	1988	562	KC699011
B	FR	1989	657	KC699009
B	FR	1997	751102	KC699024
B	FR	1998	750705	KC699020
B	FR	1999	750710	KC699021
B	GB	2008	E214_E1	HQ595776

Subtype/CRF	Country	Year	Strain or Isolate ID	Accession Number
B	GB	2008	Q230_F6	HQ595794
B	GB	2009	A626_p1	JF680906
B	GB	2009	T520b_F9	HQ595805
B	GB	1994	749CD352	AJ535607
B	JM	2009	09JM_PF0B9L	HM030565
B	JP	2011	NMC851C_clone_13	AB731669
B	KR	2002	HP_19_02LGS11_3443	KJ140264
B	KR	2008	KR012	MT021904
B	KR	2009	KOR_HIV_Env_16	KT878036
B	KR	2009	KOR_HIV_Env_17	KT878037
B	KR	2009	KOR_HIV_Env_19	KT878039
B	NL	2003	patient_A_20_3	HQ386146
B	PE	2007	502_0525_wg5	JF320191
B	PE	2013	DEMB13PE010	MH078552
B	PE	2016	DEMB16PE003	MH078554
B	PH	2015	DEMB15PH002	KY658689
B	RU	2007	RU_21_07_A9_9	HQ896488
B	SE	2012	SE600057	KP411828
B	TH	2007	AA040a_WG11	JX447156
B	TT	2000	SC46C_A4	HQ217662
B	TT	1993	QH0065_M	AF277060
B	TT	1995	QH0791_M	AF277068
B	TT	1998	TT27P_8G3	EU577148
B	UA	2001	01UAKV167	DQ823362
B	US	2000	RHPA_TF1	JN944917
B	US	2001	APV_13	DQ869019
B	US	2001	CRPE_B13	EU578061
B	US	2002	APV_16	DQ869022
B	US	2002	CR0023W	FJ469688
B	US	2002	PRLS09	FJ469758
B	US	2003	F7157	FJ469731
B	US	2003	HIV_US_BID_V5279_2003	JQ403106
B	US	2004	5417_B11	EF593190
B	US	2004	6064_10	EU578032
B	US	2004	C061711D7	KM259097
B	US	2004	CR0068P	FJ469695
B	US	2004	P070711B1	KM259452
B	US	2004	UNC2009_1	EF593269
B	US	2005	1423_F32	HQ216575
B	US	2005	306026_ENV	JX863966
B	US	2005	5791_E6	EU578004
B	US	2005	CE128FL55_28_78RNA	KX156374
B	US	2005	Z71_p1	HQ217860

Subtype/CRF	Country	Year	Strain or Isolate ID	Accession Number
B	US	2006	06US_SAJ_NVS23	JF689871
B	US	2006	502_0227_FL05	JF320036
B	US	2006	502_1512_FL01	JF320356
B	US	2006	BP00055_RH01	JN687750
B	US	2006	GETO5098_B7	HQ217266
B	US	2006	HIV_US_BID_V3047_2006	JQ403068
B	US	2007	07US_SAJ_NVS42	JF689889
B	US	2007	306349_ENV	JX864007
B	US	2007	502_0388_RH06	JF320315
B	US	2007	502_1709_04	JF320467
B	US	2007	CH341s_J8	HQ908139
B	US	2007	HIV_US_BID_V3010_2007	JQ403058
B	US	2007	P020813B10	KM259279
B	US	2008	B_US_08_470_scr_3_A5	HQ908219
B	US	2008	HIV_US_BID_V3048_2008	JQ403069
B	US	2008	HIV_US_BID_V3128_2008	JQ403082
B	US	2008	HIV_US_BID_V4120_2008	JQ403031
B	US	2009	DEMB09US003	KC473824
B	US	2010	505_0695a.WG06	MG196787
B	US	2011	505_1174a.WG06	MG196942
B	US	2011	9040_070611_CSF_1	KM354470
B	US	2011	950965	KT124809
B	US	2011	CP10_3A	KF384798
B	US	2012	409_133_F_w02VT	MH897911
B	US	2012	505_0049a.WG07	MG196653
B	US	2013	HIV_USA_AC09_post	MW924814
B	US	2013	IQA264.PBMC.SGA2	KR182173
B	US	2013	IQA276.Urine.SGA2	KR182343
B	US	2014	Pt7_DNA_5	KU678125
B	US	2015	A5340A10.w006	KX587339
B	US	2015	B155.2.d.TIT.D12.4.8.16.a8.S8	KY113566
B	US	2016	2669.LNMC.CAD.3142016.ENV_Seq1	MK148554
B	US	2016	3632_TCM_12	MN466965
B	US	2016	608_D14_MI10	MH263144
B	US	2017	00937_PH389_W1.2_3HG19...	MT307711
B	US	2017	UNC_404_PL1_J1	MW062835
B	US	2018	LFSA_LN_2G9	MK169834
B	US	2019	2990_V7_B6_2	MZ397052
B	US	2019	5257_V7_D71_A9_v2.0	MZ397107
B	US	1981	81NY3	AY247224
B	US	1983	5157_83	AY835781
B	US	1984	NY5CG	M38431
B	US	1985	5077_85	AY835769

Subtype/CRF	Country	Year	Strain or Isolate ID	Accession Number
B	US	1986	SFMHS8	AF025756
B	US	1990	US3	AY173954
B	US	1991	100307i.2.18	MH012589
B	US	1991	1744i.1.1	MT861482
B	US	1992	1039i.1.39	MT861667
B	US	1994	2564i.1.1	MT861932
B	US	1996	1304_d31	AY308762
B	US	1996	61792_03_p29	EU575474
B	US	1997	63358_p3_4013	EU289192
B	US	1998	WC3_0498_4	EF175212
B	ZA	2010	MSM304	KF726016
B	ZA	2010	MSM306	KF726018
C	BI	1991	BU910213	U39237
C	BR	2002	02BR2022	JN692434
C	BR	2004	04BR013	AY727522
C	BW	2000	00BW18113	AF443098
C	BW	2000	00BW3891_6	AF443113
C	BW	2010	mpp_00205_amp2	KR861326
C	BW	2010	mpp_00241_amp2	KR861329
C	BW	2011	mpp_00160_amp2	KR861322
C	BW	2011	mpp_00454_amp2	KR861342
C	BW	2012	mpp_00037_amp2	KR861311
C	BW	2013	mpp_00238_amp2	KR861328
C	BW	2015	074_A_110_1_4_w0_204	MK457968
C	BW	1996	96BW15B03	AF110973
C	BW	1996	96BWMO1_5	AF443074
C	BW	1998	98BWMO37D5	AF443082
C	CY	2006	CY176	FJ388952
C	CY	2008	CY219	JF683768
C	ET	2008	ET106	KU319529
C	ET	1986	ETH2220	U46016
C	FI	1992	FIN9210	AF219266
C	GB	2008	M320d_F9	HQ595787
C	GB	2008	M520c_B9	HQ595789
C	IL	1999	99ET14	AY255825
C	IL	1999	99ET7	AY255824
C	IN	2000	DEMC00IN007	KP109482
C	IN	2011	AIIMS_619_11_P1	MK521805
C	IN	2017	AIIMS731_73105b	MT366192
C	IN	2018	AIIMS743_74311A	MN703391
C	IN	1993	93IN999	AF067154
C	IN	1994	94IN476	AF286223
C	IN	1995	95IN21068	AF067155

Subtype/CRF	Country	Year	Strain or Isolate ID	Accession Number
C	IN	1998	98IN012	AF286231
C	KE	2005	05KE369195V4	KT022371
C	KE	2011	093_KE	KU921723
C	MM	1999	mIDU101_3	AB097871
C	MW	2003	0682_E4	KC894077
C	MW	2005	3025_2_C3	KC894092
C	MW	2005	3026_2_H9	KC894093
C	MW	2006	2052_G10	KC894084
C	MW	2007	DEMC07MW001	KP109520
C	MW	2008	703010588_E7	HQ615963
C	MW	2009	3009_Plasma_Visit_1_amplicon6	KC186690
C	MW	2009	4039_Plasma_Visit1_amplicon12	KC187620
C	MW	1996	S031_B2_A6	MT227426
C	NP	2011	11NP007	KJ158423
C	NP	2015	NP13	MK493076
C	SE	2008	SE600213	KP411834
C	SE	2010	043SE	MF373154
C	SE	2012	073NP	MF373176
C	SE	2014	097SO	MF373199
C	TZ	2000	410_F2_7	HQ698009
C	TZ	2001	A207	AY253307
C	TZ	2001	BD16_10	AY253320
C	TZ	2002	CO6721	AY734560
C	UG	1990	UG268A2	L22948
C	YE	2002	02YE511	AY795906
C	ZA	2003	03ZAPS077B1	DQ093591
C	ZA	2003	03ZAPS094MB1	DQ396377
C	ZA	2003	03ZAPS104MB1	DQ369990
C	ZA	2003	03ZASK019B2	AY878063
C	ZA	2003	03ZASK073B1	AY901970
C	ZA	2003	04ZAPS194MB1	DQ275650
C	ZA	2003	SK040B1	AY703908
C	ZA	2004	04ZAPS172MB1	DQ275659
C	ZA	2004	04ZAPS177MB1	DQ396394
C	ZA	2004	04ZAPS217B1	DQ164119
C	ZA	2004	04ZASK181B1	AY878062
C	ZA	2005	05ZAFV15	DQ382373
C	ZA	2005	05ZAFV23	DQ382376
C	ZA	2005	05ZASK243B1	DQ396372
C	ZA	2005	05ZASK247B1	DQ369994
C	ZA	2006	CHV0005480_CAP69.1.12TA8	FJ443300
C	ZA	2006	CHV0006062_CAP260.2.00G11	FJ443488
C	ZA	2007	2935054	HQ595758

Subtype/CRF	Country	Year	Strain or Isolate ID	Accession Number
C	ZA	2007	3514597	HQ595759
C	ZA	2007	503_09003_5	KT183229
C	ZA	2007	503_10430_2	KT183253
C	ZA	2007	704010042_CH042_TF	KC156123
C	ZA	2007	704010124_E6	KC894117
C	ZA	2008	705010534_CH534.w12	KC156221
C	ZA	2008	707PKE05F1	HM623551
C	ZA	2008	707PKE18F2	HM623562
C	ZA	2008	CAP311_2_00_G5_1	KC154021
C	ZA	2008	DEMC08ZA011	JX140666
C	ZA	2009	21369737_G11_F2	HQ615941
C	ZA	2009	21399975_E2_B3	HQ615957
C	ZA	2009	503_05130_2	KT183141
C	ZA	2009	704MC024N	GU080187
C	ZA	2009	707PKE21N1	HM623602
C	ZA	2009	CM050C_SGA05	MF284967
C	ZA	2009	TRP347_2_00_B1_1	JN681258
C	ZA	2009	TRP363_2_00_10_3	JN681259
C	ZA	2010	MSM150	KF725935
C	ZA	2010	MSM218	KF725957
C	ZA	2010	MSM248	KF725976
C	ZA	2014	DEMC14ZA176	MT222958
C	ZA	2014	ZA006EP1C7	MN611462
C	ZA	2015	Pt1.1_S1052	MK643538
C	ZA	2016	CAP188_B_W31	MN097551
C	ZA	2017	CAP287_W13	MN097648
C	ZA	1989	pZAC_R3714	JN188292
C	ZA	1997	97ZA009	AY118166
C	ZM	2003	ZM246F_flD5	FJ496194
C	ZM	2004	23B.PB.3148	KY229650
C	ZM	2005	ZM406_200406_26	GU329333
C	ZM	2007	ZM326M_17Feb2007_SC_10_N	MT195232
C	ZM	2009	DEMC09ZM015	KU749426
C	ZM	2009	Z3576M_18Apr09_PL_NFLG_SGA10	KR820326
D	BR	2010	10BR_RJ108	KJ787683
D	CD	2002	LA18ZiAn	KU168272
D	CD	2003	LA17MuBo	KU168271
D	CD	1984	84ZR085	U88822
D	CD	1987	PBS5635	MH705152
D	CM	2001	01CM_0175BA	AY371156
D	CM	2001	01CM_4412HAL	AY371157
D	ES	2009	P2351	JN054274
D	FI	1993	FIN93178	AF219272

Subtype/CRF	Country	Year	Strain or Isolate ID	Accession Number
D	GM	1994	N73603	HQ385448
D	KE	1997	QB857_110I_ENV_B3	FJ866138
D	SN	1990	SE365A2	L22945
D	TZ	2000	54_F4_A10	HQ659623
D	TZ	2001	A280	AY253311
D	TZ	2004	CO6405V4	KX907406
D	UG	2005	2810MP1	KF986131
D	UG	2006	927MP1	KF986041
D	UG	2007	192002	MW006058
D	UG	2007	9009SA_A4_2	HM215351
D	UG	2008	270535	MW006078
D	UG	2010	DEMD10UG004	KF716479
D	UG	2011	194535	MW006073
D	UG	1991	UG270	AB485651
D	UG	1992	92UG001	AJ320484
D	UG	1995	42_877	MH705143
D	UG	1996	A03836B1_6	JX658585
D	UG	1997	338MPc01	EU853046
D	UG	1998	372FPc02	EU853078
D	UG	1998	98UG57131	AF484505
D	UG	1999	99UGB25647	AF484481
D	UG	1999	99UGD26830	AF484486
D	UG	1999	99UGE08364	AF484487
D	UG	1999	99UGE13613	AF484515
D	UG	1999	99UGG35093	AF484495
D	ZA	1985	R286	AY773340
D	ZA	1986	R482	AY773341
D	ZM	2005	ZM387_200387_5	GU329179
F1	AO	2006	AO_06_ANG32	FJ900266
F1	BE	1994	14_00_37	DQ313239
F1	BG	2009	S_09_155	MH746241
F1	BR	2001	01BRRJSB153	MG365763
F1	BR	2002	02BR082	FJ771006
F1	BR	2006	06BR564	FJ771008
F1	BR	2007	07BR844	FJ771010
F1	BR	2008	08BRRJ35	MG365767
F1	BR	2010	10BR_RJ055	KT427774
F1	BR	2010	10BR_RJ084_2	KT427868
F1	BR	2011	11BRRJPR90	MG365768
F1	BR	2012	12BRRJP05	MG365762
F1	BR	2012	12BRRJPR51	MG365764
F1	BR	2012	2012BRRJNEUT39	KX181922
F1	BR	2012	2012BRRJNEUT43	KX181926

Subtype/CRF	Country	Year	Strain or Isolate ID	Accession Number
F1	BR	2013	2013BRRJNEUT31	KX181917
F1	BR	2013	2013BRRJNEUT35	KX181919
F1	BR	1993	93BR020_1	AF005494
F1	ES	2008	X2674_C4	HQ236616
F1	ES	2009	X2687_f3	HM068551
F1	ES	2011	VA0053_nfl	KJ883138
F1	ES	2014	100347	MF381271
F1	ES	2016	P4443_C3	MW584222
F1	FI	1993	FIN9363	AF075703
F1	FR	2004	LA22LeRe	KU168276
F1	FR	1996	PHI420	AY231157
F1	x	2003	LA21LeAn	KU168275
F2	CM	2001	A1699	MH705144
F2	CM	2002	02CM_0016BBY	AY371158
F2	CM	2008	08CMBDSH132	MN153483
F2	CM	2008	08CMBDSH24	MN153485
F2	CM	2010	DEMF210CM001	JX140672
F2	CM	2010	DEMF210CM007	JX140673
F2	CM	2011	DEMF211CM025	KU749420
F2	CM	1993	CA20	AJ277824
F2	CM	1995	95CM_MP255	AJ249236
F2	CM	1995	95CM_MP257	AJ249237
F2	CM	1997	CM53657	AF377956
F2	ES	2008	P2059_a_B3	JN054264
F2	ZA	2010	MSM237	KF725967
G	BE	2008	CV	MN486044
G	BE	1996	DRCBL	AF084936
G	BG	2009	S_09_025	MH746233
G	BG	2009	S_09_122	MH746240
G	BG	2009	V_09_011	MH746255
G	CD	2003	LA23LiEd	KU168277
G	CD	1987	87_2580	MH705162
G	CD	1987	P406	MH705155
G	CD	1987	PBS1191	MH705134
G	CM	2001	01CM_4049HAN	AY371121
G	CM	2001	A1786	FJ389367
G	CM	2003	CM44_10	KU168302
G	CM	2004	178_15	FJ389363
G	CM	2004	314_40	FJ389364
G	CM	2004	515_28	FJ389365
G	CM	2004	944_5	FJ389366
G	CM	2005	05CMBDSH19	MN153484
G	CM	2005	144_26	MH705145

Subtype/CRF	Country	Year	Strain or Isolate ID	Accession Number
G	CM	2006	740_14	KP718915
G	CM	2007	920_49	KP718923
G	CM	2007	BS03_A1_01062011	KR051438
G	CM	2007	BS12_A5_21032012	KR051442
G	CM	2007	BS46_A2_21032012	KR051448
G	CM	2007	BS48	KR017776
G	CM	2007	BS51_A3_21032012	KR051457
G	CM	2008	789_10	KP718925
G	CM	2009	10056_C12_31022012	KR051418
G	CM	2009	11439_C5	KR051426
G	CM	2010	10CMLB030	MT349406
G	CM	2010	10CMLB040	MT349407
G	CM	2010	12541_B2	KR051432
G	CM	2010	DEMG10CM008	JX140676
G	CM	2010	DEURF10CM020	KP109502
G	CM	2011	DEMG11CM046	KY658701
G	CM	1996	96CMABB55	AY772535
G	CM	1997	97CM_MP801	AM279346
G	CM	1998	98CM_MP1033	AM279365
G	CM	1999	99CM_MP1287	AM279351
G	CM	1999	99CM_MP1416	AM279359
G	CM	1999	99CM_MP1417	AM279350
G	CN	2006	sh52	HM067749
G	CN	2008	GX_2084_08	JN106043
G	CN	2013	GZ8H3748.13	MH431770
G	CN	2016	10818	MK254637
G	CN	2016	224GX	KY275364
G	CN	2016	9835	MK254639
G	CU	1999	Cu74	AY586547
G	CU	1999	Cu85	AY586548
G	CU	1999	Cu87	AY586549
G	ES	2000	X558	AF423760
G	ES	2002	P402_2_11	EU885759
G	ES	2002	X1193_1	EU885761
G	ES	2003	X1254_3	EU885762
G	ES	2004	X1632_s2_b10	FJ817370
G	ES	2005	P962	EU786670
G	ES	2005	X1628_2	FJ670520
G	ES	2005	X1854_2_10	EU885763
G	ES	2005	X2131_c1_B5	FJ817368
G	ES	2006	X2088_9	EU885764
G	ES	2007	MFU54_D1	HQ236564
G	ES	2007	X2160_r25	EU885765

Subtype/CRF	Country	Year	Strain or Isolate ID	Accession Number
G	ES	2007	X2234_H11	HQ236608
G	ES	2008	P1909_C5_6	GU332513
G	ES	2008	P1981_2	FJ670530
G	ES	2008	P2091_a	JN054265
G	ES	2008	X2470_F8_12	GQ222685
G	ES	2008	X2483_F12_3	GQ222686
G	ES	2008	X2558	GQ862781
G	ES	2008	X2571_b1	JF327808
G	ES	2009	P2272_b	JN054269
G	ES	2009	X2634_2	GU362882
G	ES	2009	X2634_a	JN054285
G	ES	2009	X2636_b	JN054287
G	ES	2009	X2644_b	JN054289
G	ES	2009	X2693_a	JN054296
G	ES	2014	ARP1201	KT276261
G	ES	2014	EUR_0033	KU685592
G	ES	1999	X138	AF450098
G	FR	1995	PHI355	AY231155
G	FR	1995	PHI365	AY231156
G	GH	2020	KBH16_GH	OQ121858
G	GH	2020	KBH36_GH	OQ121873
G	GH	2021	KBH86_GH	OQ121909
G	GW	2008	LA57LmNe	KU168300
G	KE	2006	06KE275457V6	KT022379
G	KE	2009	DEMG09KE001	KF716477
G	KE	2017	V703_0865_070_RE_con_s	ON890999
G	KE	1993	HH8793	AB485662
G	NG	2001	01NGPL0669	DQ168576
G	NG	2001	01NGPL0674	DQ168575
G	NG	2001	01NGPL0760	DQ168579
G	NG	2001	PL0567	DQ168573
G	NG	2008	08NG_SC13	JN248582
G	NG	2008	P1909_A12	HQ236581
G	NG	2008	P1992_G10_4	GQ324613
G	NG	2009	09NG010079	KX389636
G	NG	2009	09NG010105	KX389635
G	NG	2009	09NG010157	KX389631
G	NG	2009	09NG010205	KX389628
G	NG	2009	09NG010261	KX389626
G	NG	2009	09NG010315	KX389625
G	NG	2009	09NG_SC21	JN248584
G	NG	2009	09NG_SC26	JN248586
G	NG	2009	09NG_SC31	JN248591

Subtype/CRF	Country	Year	Strain or Isolate ID	Accession Number
G	NG	2009	09NG_SC62	JN248593
G	NG	2010	10NG020133	KX389620
G	NG	2010	10NG020134	KX389619
G	NG	2010	10NG020303	KX389618
G	NG	2010	10NG020420	KX389615
G	NG	2011	11NG050158	KX389641
G	NG	2011	11NG050272	KX389642
G	NG	2011	11NG050489	KX389645
G	NG	2011	DEMG11NG006	KY953200
G	NG	2011	DEMG11NG007	KY953201
G	NG	2011	DEMG11NG008	KY953202
G	NG	2012	12NG060248	KX389646
G	NG	2012	12NG060409	KX389648
G	NG	2015	AC02_A5_HIV_1G	MN944109
G	NG	2015	AC04_A2_HIV_1G	MN944135
G	NG	2015	AC05_A3_HIV_1G	MN944150
G	NG	2015	AC13A_A2_HIV_1G	MN944231
G	NG	1992	92NG083_JV10832	U88826
G	NG	1995	NG1928	AF069947
G	NG	1995	NG1929	AF069943
G	NG	1995	NG1937	AF069937
G	NG	1995	NG1939	AF069935
G	RU	2012	RU_SRD_2012	MF614606
G	RU	1989	RU_ShRMT_1989	MF614605
G	SE	1993	SE6165_G6165	AF061642
G	US	2016	00926_PH331_W2_DMSO...	MT307670
G	ZA	2001	TV546	KJ948662
G	ZM	2003	8B.PB.103	KY229519
G	ZM	2003	8M.PB.203	KY229533
G	x	2003	LA24HoCe	KU168278
G	x	2006	X558_8_B11	HQ236619
G	x	2008	X2486_F2	HQ236615
H	BE	2006	CX	MN486046
H	BE	1993	VI991	AF190127
H	BE	1993	VI997	AF190128
H	CD	2001	CG_0260_02V_NGSID16	KY392779
H	CD	2001	CG_0536_02_NGSID14	KY392777
H	CD	2001	CG_0538_02_NGSID15	KY392778
H	CD	2004	LA19KoSa	KU168273
H	CF	2002	LA25LeMi	KU168279
H	CF	1990	56	AF005496
H	GB	2000	00GBAC4001	FJ711703
J	AO	1993	93AOHDC253	KU310620

Subtype/CRF	Country	Year	Strain or Isolate ID	Accession Number
J	CD	2002	CG_0331_02V_NGSID13	KY392776
J	CD	2003	LA26DiAn	KU168280
J	CD	1997	J_97DC_KTB147	EF614151
J	CM	2004	04CMU11421	GU237072
J	SE	1993	SE9280_7887	AF082394
J	SE	1994	SE9173_7022	AF082395
K	CD	1997	97ZR_EQTB11	AJ249235
K	CM	1996	96CM_MP535	AJ249239
L	CD	2001	L_CG_0018a_01	MN271384
L	CD	1983	83CD003_Z3	AF286236
L	CD	1990	90CD121E12	AF457101

Supplemental Figure S1: Maximum likelihood tree of DRC specimens along with 467 Genbank references.

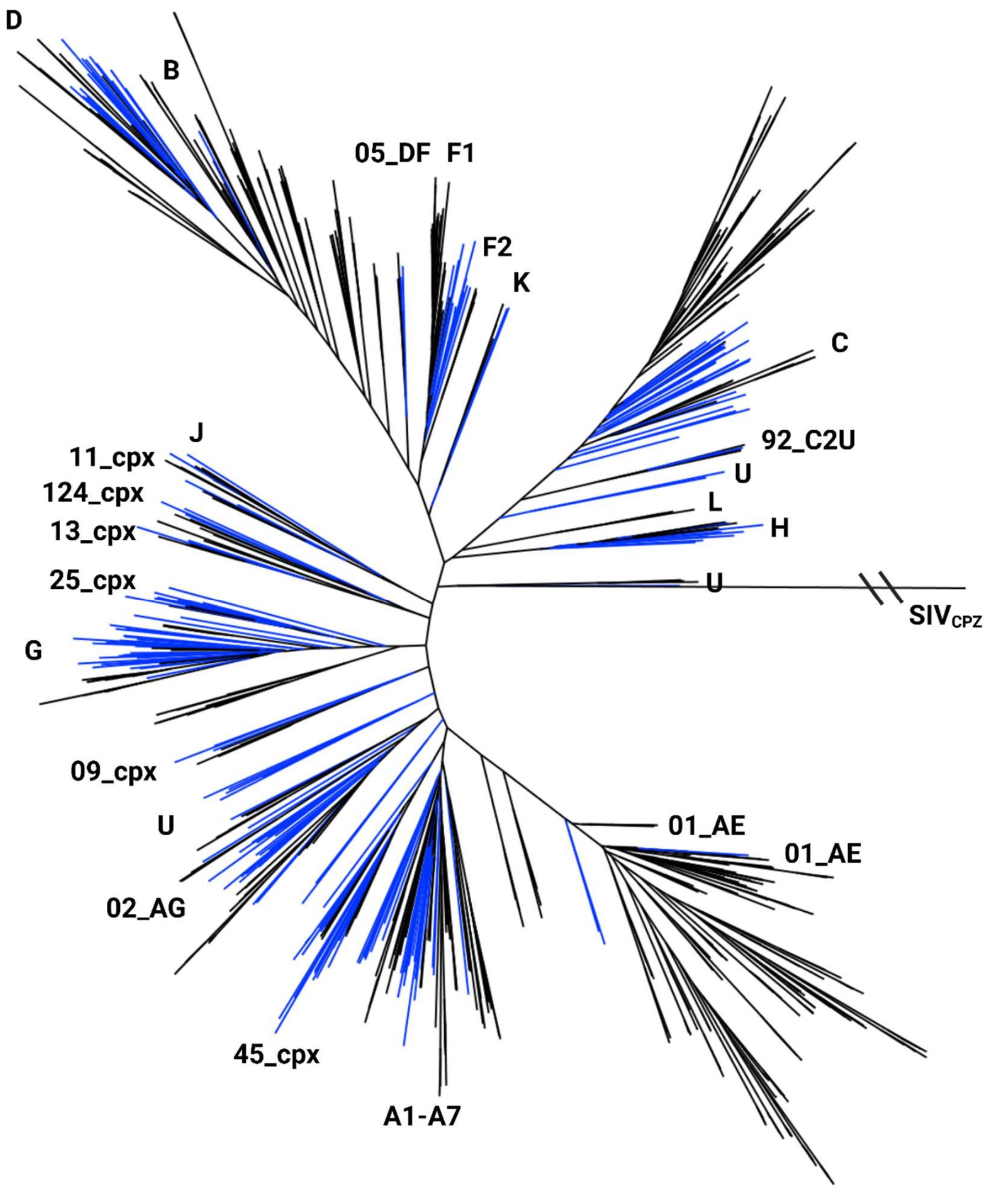
D
B
05_DF
F1
F2
K
C
92_C2U
U
L
H
U
SIV CPZ
J
11_cpx
124_cpx
13_cpx
25_cpx
G
09_cpx
U
02_AG
45_cpx
A1-A7
01_AE
01_AE

Supplemental Figure S2: Maximum-likelihood phylogeny performed via the Augur pipeline (NextStrain), zoomed to the branch of subtype B of which the HIV-1 full genome from 18CD-0068 belongs. (A) Fully phylogeny with the subtype B clade of interest shaded in red. (B) A zoomed view of the clade of interest. (C) A table showing the collection location of the top 100 BLAST hits to the full genome of isolate 18CD-0068.

A

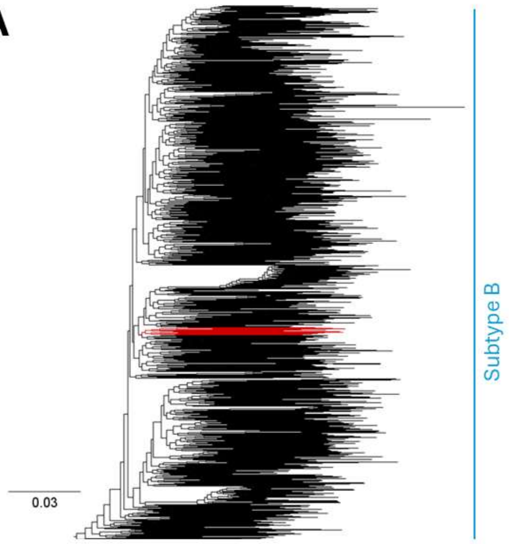

B

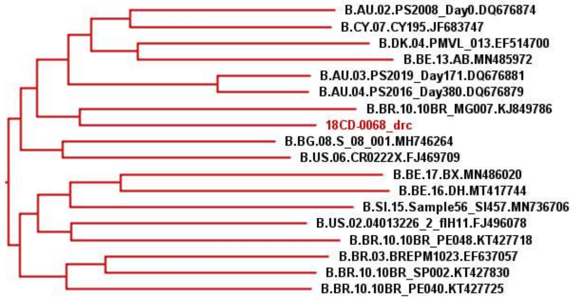

C

Collection location of top 100 BLAST hits

Country	Count
United States	37
Not listed	27
Spain	15
South Korea	13
France	5
Netherlands	2
Brazil	1

Supplemental Figure S3: Comparison of results obtained from RealTi*m*e HIV-1 (x-axis) and Alinity m viral load (y-axis) testing. A total of 161 specimens had sufficient volume for comparison testing. R^2 was calculated in GraphPad Prism.

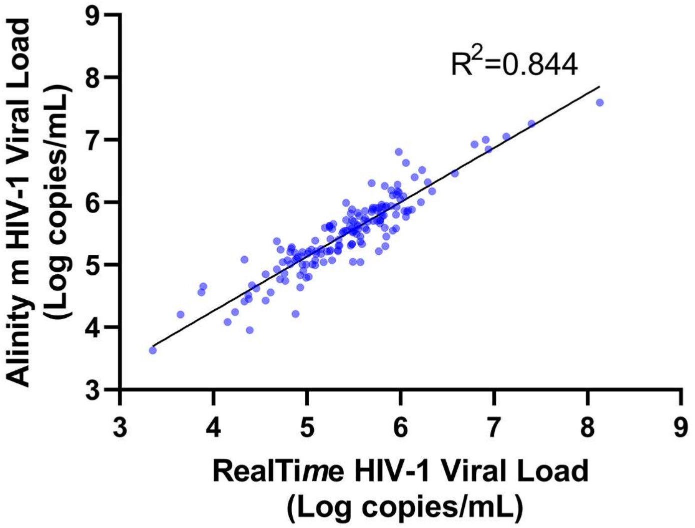

Supplemental Figure S4: Maximum likelihood-based strict molecular clock analysis (root-to-tip distance versus sampling date using the NextStrain pipeline) of HIV-1 *env* sequences from the LANL reference dataset alone (A) and the LANL reference dataset combined with the 197 new sequences recovered in the current study (B). Linear regressions with accompanying clock rate estimates are shown for sequences from all countries (including DRC; blue) and the DRC alone (orange).

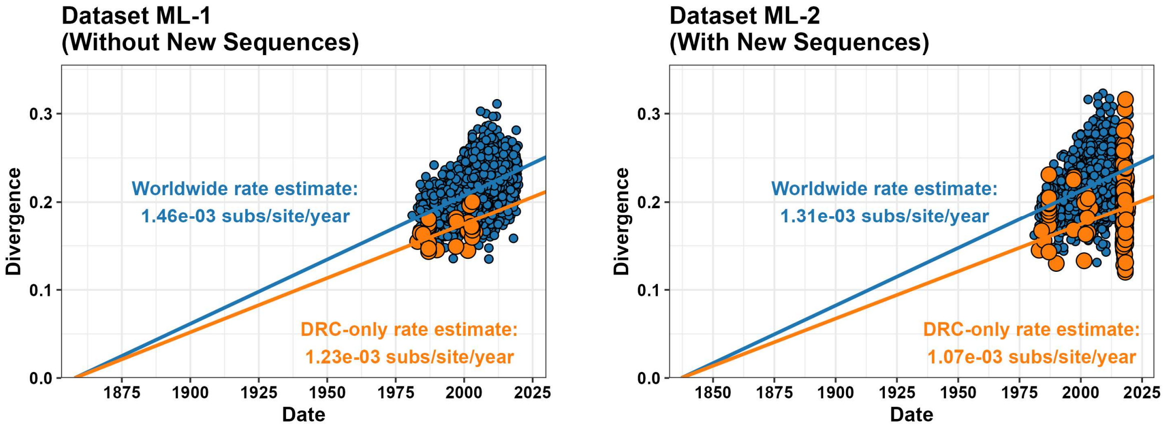

Supplemental Figure S5: SimPlot and bootscan of 8 additional subtype H specimens identified in the study. A window of 500 bp and step of 50 bp was used for all analyses. For bootscanning 1000 bootstrap replicates was used. Subtype reference consensus sequences were used for SimPlot and Bootscan analyses.

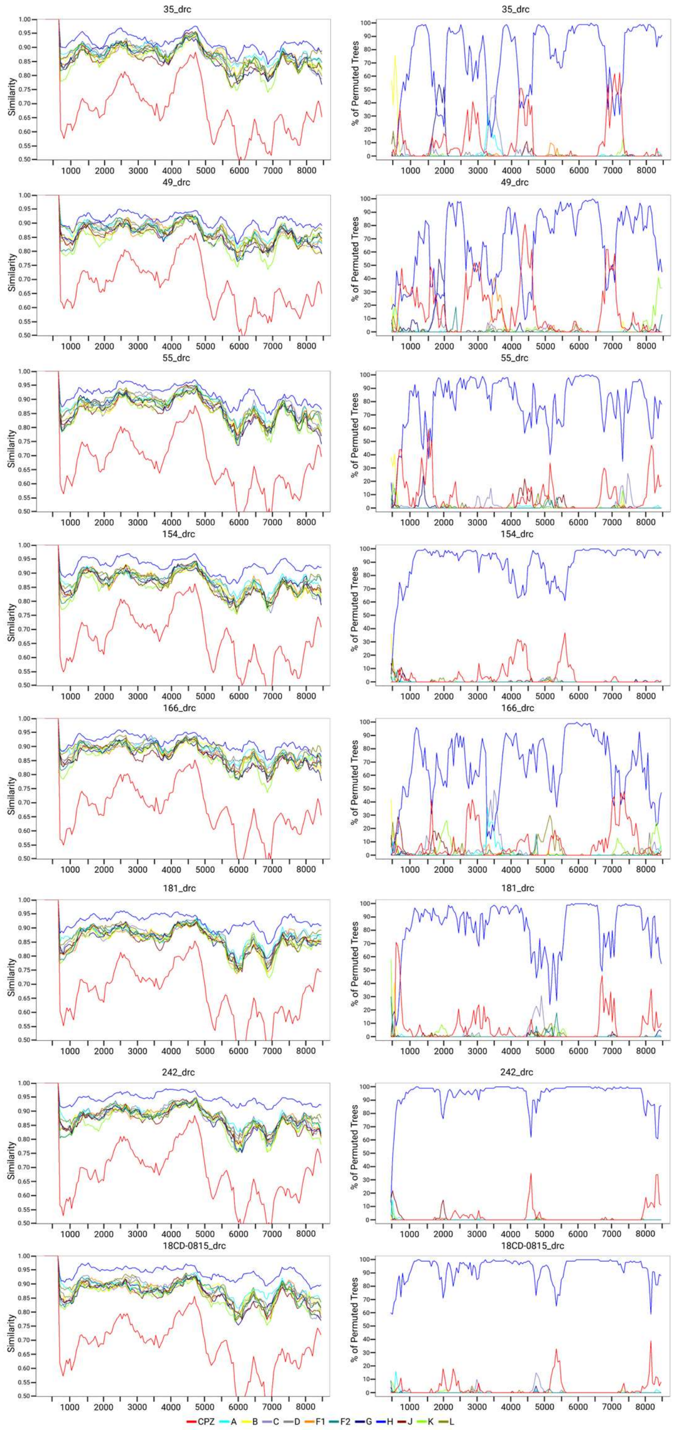

Supplement: Supplementary file 1 [file viruses-18-00268-s001.zip › viruses-4151694-supplementary.pdf]
